# Development and Validation of Patient Education Tools for Deprescribing in Patients on Hemodialysis

**DOI:** 10.1177/20543581221150676

**Published:** 2023-01-24

**Authors:** Thomas Hyunwoo Cho, Patrick C. K. Ng, Melissa J. Lefebvre, Arlene Desjarlais, Dennis McCann, Blair Waldvogel, Marcello Tonelli, Amit X. Garg, JoAnne Wilson, Monica Beaulieu, Judith Marin, Cali Orsulak, Melanie Talson, Monica Sharma, Jordanne Feldberg, Clara Bohm, Marisa Battistella

**Affiliations:** 1 Department of Pharmacy, University Health Network, Toronto, ON, Canada; 2 Patient Partners, Can-SOLVE CKD Network, Vancouver, BC, Canada; 3 University of Calgary, AB, Canada; 4 Institute for Clinical Evaluative Sciences, Toronto, ON, Canada; 5 Division of Nephrology, Department of Medicine, Epidemiology and Biostatistics, Western University, London, ON, Canada; 6 Division of Nephrology, Department of Medicine, Nova Scotia Health Authority, Halifax, Canada; 7 Faculty of Health, Dalhousie University, Halifax, NS, Canada; 8 Division of Nephrology, The University of British Columbia, Vancouver, Canada; 9 Providence Health Care, Vancouver, BC, Canada; 10 Health Sciences Centre Winnipeg, MB, Canada; 11 Department of Internal Medicine, Max Rady College of Medicine, University of Manitoba, Winnipeg, Canada; 12 Leslie Dan Faculty of Pharmacy, University of Toronto, ON, Canada

**Keywords:** deprescribing, hemodialysis (HD), validation, patient education, shared decision-making

## Abstract

**Background:**

Deprescribing is a patient-centered solution to reducing polypharmacy in patients on hemodialysis (HD). In a deprescribing pilot study, patients were hesitant to participate due to limited understanding of their own medications and their unfamiliarity with the concept of deprescribing. Therefore, patient education materials designed to address these knowledge gaps can overcome barriers to shared decision-making and reduce hesitancy regarding deprescribing.

**Objective:**

To develop and validate a medication-specific, patient education toolkit (bulletin and video) that will supplement an upcoming nationwide deprescribing program for patients on HD.

**Methods:**

Patient education tools were developed based on the content of previously validated deprescribing algorithms and literature searches for patients’ preferences in education. A preliminary round of validation was completed by 5 clinicians to provide feedback on the accuracy and clarity of the education tools. Then, 3 validation rounds were completed by patients on HD across 3 sites in Vancouver, Winnipeg, and Toronto. Content and face validity were evaluated on a 4-point and 5-point Likert scale, respectively. The content validity index (CVI) score was calculated after each round, and revisions were made based on patient feedback.

**Results:**

A total of 105 patients participated in the validation. All 10 education tools achieved content and face validity after 3 rounds. The CVI score was 1.0 for most of the tools, with 0.95 being the lowest value. Face validity ranged from 72% to 100%, with majority scoring above 90%.

**Conclusion:**

Ten patient education tools on deprescribing were developed and validated by patients on HD. These validated, medication-specific education tools are the first of its kind for patients on HD and will be used in a nationwide implementation study alongside the validated deprescribing algorithms developed by our research group.

## Abrégé

**Contexte:**

La déprescription est une solution axée sur le patient pour réduire la polypharmacie chez les patients sous hémodialyse (HD). Dans une étude pilote sur la déprescription, les patients ont hésité à participer en raison de leur compréhension limitée de leurs propres médicaments et de leur manque de connaissance du concept de déprescription. Par conséquent, du matériel éducatif conçu pour combler ces lacunes dans les connaissances des patients pourrait surmonter les obstacles à la prise de décision partagée et réduire les hésitations à l’égard de la déprescription.

**Objectifs:**

Développer et valider une trousse d’information (bulletin et vidéo) pour les patients portant sur les médicaments. Cette trousse viendra compléter un futur programme national de déprescription pour les patients sous HD.

**Méthodologie:**

Des outils d’éducation pour les patients ont été développés à partir du contenu d’algorithmes de déprescription validés précédemment et de recherches documentaires sur les préférences des patients en matière d’éducation. Une ronde préliminaire de validation a été complétée par cinq cliniciens afin d’obtenir des commentaires sur l’exactitude et la clarté des outils d’éducation. Trois cycles de validation ont ensuite été réalisés par des patients sous HD dans trois sites: Vancouver, Winnipeg et Toronto. La validité du contenu et la validité apparente ont été évaluées à l’aide d’échelles de Likert à 4 et 5 points, respectivement. L’indice de validité du contenu (IVC) a été calculé après chaque ronde et des révisions ont été effectuées en fonction des commentaires des patients.

**Résultats:**

En tout, 105 patients ont participé à la validation. La validité du contenu et la validité apparente ont été atteintes pour les dix outils d’éducation après trois rondes auprès des patients. L’IVC s’établissait à 1,0 pour la plupart des outils évalués; 0,95 était la valeur d’indice la plus faible. La validité apparente variait entre 72% et 100%, la majorité des outils ayant obtenu un score supérieur à 90%.

**Conclusion:**

Dix outils d’éducation pour les patients portant sur la déprescription ont été développés et validés par des patients sous HD. Ces outils d’éducation validés portant spécifiquement sur les médicaments sont les premiers du genre conçus pour les patients sous HD. Ils seront utilisés dans le cadre d’une étude nationale de mise en œuvre, parallèlement aux algorithmes validés de déprescription qui ont été développés par notre groupe de recherche.

## Introduction

Polypharmacy is common in patients on hemodialysis (HD) due to the presence of comorbidities that require long-term medication management (eg, diabetes, cardiovascular disease, hypertension).^1-3^ Polypharmacy is associated with an increased risk of adverse drug reactions (ADRs) such as falls, functional and cognitive decline, drug-drug interactions, and mortality.^4-7^ These risks are further exacerbated by the use of potentially inappropriate medications (defined as medications in which the potential risk of occurrence of ADRs may be greater than the clinical benefit), adding to the patient’s polypharmacy.^8,9^ Moreover, due to altered pharmacokinetics in end-stage kidney disease, the potential for adverse outcomes increases with the use of additional potentially inappropriate medications.^9-11^

Deprescribing, “the planned and supervised process of discontinuing medications that may be causing harm or are no longer providing benefit,” is a potentially safe and effective solution to reducing the use of inappropriate medications.^12,13^ In particular, tool-guided deprescribing strategies have demonstrated their potential for minimizing the adverse risks of polypharmacy and contributing toward patient-centered goals of care.^13-17^ Deprescribing tools for clinicians are available but often developed for the elderly, not medication-specific, and do not meet the specific needs of patients on HD.^13^ To address this, we validated 9 medication-specific deprescribing algorithms (alpha-1 blockers, benzodiazepines and Z-medications [eg, zopiclone], gabapentinoids, loop diuretics, prokinetic agents, proton pump inhibitors, quinine, statins, and urate-lowering agents) for HD clinicians to meet and account for HD-specific needs.^13^ We previously completed a successful single-center deprescribing pilot study in patients on HD using these validated algorithms, where patients stayed successfully deprescribed after 6 months without any observed adverse events.^15^ While our deprescribing algorithms follow demands for validated clinical decision-making tools, our experience in conducting the pilot study revealed that patient barriers toward a successful real-world implementation of these algorithms persist.^13,18^

Throughout our deprescribing pilot study, patients expressed difficulty recalling original indications for medications, hesitancy toward medication changes, and unfamiliarity with the concept of deprescribing. These apprehensions were often eased during the consent discussion—patients desired communication about their medications and welcomed the additional involvement in their care. This follows previously identified patient perspectives on shared decision-making (SDM) and patient barriers to deprescribing.^18,19^ Patients may want to be involved in their own care but lack health literacy and knowledge about their condition and medications, greatly influencing the extent of their involvement and information seeking.^19,20^ A *patient-centered* deprescribing intervention crucially involves SDM, which necessitates the patient to be willing and able to engage in the deliberation process.^19,21^ In turn, this intervention should address the observed patient barriers of fear (“Will my disease worsen?”), appropriateness (“If a drug is inappropriate, why am I still taking it?”), and process (“How do I stop a drug? Will the clinical team notice negative effects in time?”) through patient education.^18,19,22^ Materials that effectively communicate lay information on medication and deprescribing can help patients engage in meaningful conversations with their provider, regardless of education and literacy levels.^22-24^ Throughout our deprescribing algorithms, patient engagement is emphasized and prompted to the clinician; however, patients may not sufficiently understand the process even after verbal discussions with their care provider.^13,18,25^ Although deprescribing has been shown in the literature to be a safe and effective approach to reducing polypharmacy, the aforementioned patient barriers have not been addressed.^20,25,26^

Patient barriers to deprescribing must be addressed to facilitate the successful and sustainable implementation of a deprescribing program.^25,26^

Supplementing treatment algorithms with patient education materials could help bolster the likelihood of successful deprescribing and increase the adoption of deprescribing practices by clinicians and patients.^25,26^

Here, we describe the development, face validation, and content validation of patient education tools for deprescribing in patients on HD. The study objectives were (1) to develop patient deprescribing education tools (a bulletin and a video) for 9 specific medication classes for patients on HD and 1 general deprescribing information tool and (2) to formally validate the tools with patients on HD for face and content validity.

## Methods

The study consisted of 2 phases: (1) development of medication-specific patient education tools consisting of a bulletin and a video (for our 9 validated algorithms: alpha-1 blockers, benzodiazepines and Z-medications [eg, zopiclone], gabapentinoids, loop diuretics, prokinetic agents, proton pump inhibitors, quinine, statins, and urate-lowering agents) and (2) validation of the patient education tools for content and face validity using the Lynn method.^27^

### Phase 1: Development of the Patient Education Tools

Literature searches using MEDLINE (1990 to September 1, 2017) and CINAHL (1990 to September 1, 2017) were conducted with a librarian to identify (1) patients’ preferences on drug information in patient educational materials and (2) existing information on deprescribing in patient education materials (keywords listed in Appendix I-A).

Bulletins and videos were created in Microsoft Publisher (Redmond, Washington) and VideoScribe (Sparkol, Bristol, United Kingdom), respectively. The content included in each medication-specific tool was a synthesis of literature search results and relevant drug information in the respective deprescribing algorithm. A librarian reviewed all materials for lay-appropriate language before undergoing the validation process.

### Phase 2: Content and Face Validation

Validation was based on the Lynn method and consistent with the methods used for our deprescribing algorithms.^13,27^ The Lynn method requires a minimum of 5 unique reviewers to control for chance agreement; thus, 6 patients were recruited to validate each tool.^27^ There is no optimum number of rounds for building consensus; a minimum of 3 rounds were performed with revisions of the education tools based on participants’ feedback between each round.^28^

A preliminary validation round was completed with 5 nephrology clinicians at Toronto General Hospital (Toronto, Ontario, Canada) and 3 project patient partners (Can-SOLVE CKD Patient Council; Vancouver, British Columbia, Canada). Next, 3 patient validation rounds took place across 3 Canadian study sites: (1) Toronto (Toronto General Hospital), (2) Winnipeg (Seven Oaks General Hospital, Winnipeg, Manitoba, Canada), and (3) Vancouver (St. Paul’s Hospital, Vancouver, British Columbia, Canada). In Winnipeg and Vancouver, a team of 1 research assistant and 2 patient research partners conducted half of all the validation interviews for rounds 1 and 2, while round 3 was completed solely by the research assistants due to hospital restrictions during the COVID-19 pandemic. In Toronto, a research assistant conducted all interviews. This study was approved by the University Health Network Research Ethics Board (REB; Toronto) (ID: 17-5313); Winnipeg REB (HS21985); and Vancouver REB (ID: H18-01480-A007).

Each patient education tool was reviewed by 6 patients per round through one-on-one interviews, except for prokinetic agents, which were reviewed by 5 patients per round. Patients received a copy of the bulletin and a 2-part questionnaire and were provided with a device (Apple iPad; Cupertino, California) to view the video during the validation interviews. Patients were included based on 3 criteria at the time of approach: willingness to participate, taking at least 5 active medications, and being on HD for 3 months or longer. Patients unable to understand English or with dementia were excluded.

#### Questionnaires for validation process

Content and face validity questionnaires were based on Feinstein’s concept of clinical sensibility and the Agency for Healthcare Research and Quality’s Patient Education Materials Assessment Tool for Printable and Audio-Visual Materials.^29,30^

##### Part A (content validity)

Participants assessed individual components of the bulletin and video using a 4-point Likert scale where 1 = irrelevant and 4 = extremely relevant (Appendix I-B). Participants were asked to provide comments where revisions were thought necessary. Components rated 2 or lower by 1 or more participants required revision.^27^

##### Part B (face validity)

Participants assessed the video and bulletin overall by rating a series of statements assessing face validity on a 5-point scale (1 = strongly disagree to 5 = strongly agree) (Appendix I-B). Components rated 3 or lower by 1 or more participants required revision.

### Statistical Analysis

#### Part A (content validity)

Content validity was quantified by calculating the content validity index (CVI) score for each individual component. A component scoring 3 (acceptable with minor revision) or higher was deemed content valid; components scoring 2 (unacceptable—major revision required) or lower were revised. CVI scores per component were calculated by the proportion of participants ranking the component as valid. The overall CVI score for bulletin or video was calculated by averaging the component CVI scores within each bulletin or video. In a panel of 5 reviewers, 100% agreement (score of 3 or higher) is needed to establish content validity at the *P* < .05 level.^27^ A minimum value of 80% is cited in literature as the threshold for validity.^31^ Components ranked invalid were either revised for the next round or discarded.

#### Part B (face validity)

Face validity was reported as a percentage of study participants rating statements with a score of 4 or higher (agree, strongly agree). Face validity is a subjective measure without a standard threshold for agreement; a common threshold of ≥70% for published studies using the Delphi technique was adopted.^32,33^

### Postvalidation Literature Search

After the tools were developed and validated, an updated literature search was performed using identical search terms on MEDLINE (2017 to August 5, 2022) and CINAHL (2017 to August 5, 2022) to ensure the relevancy of the patient education tools as the validation process took longer than anticipated because of COVID-19 interruptions in the study procedures.

## Results

### Phase 1: Development of the Patient Education Tools

**Figure 1.**
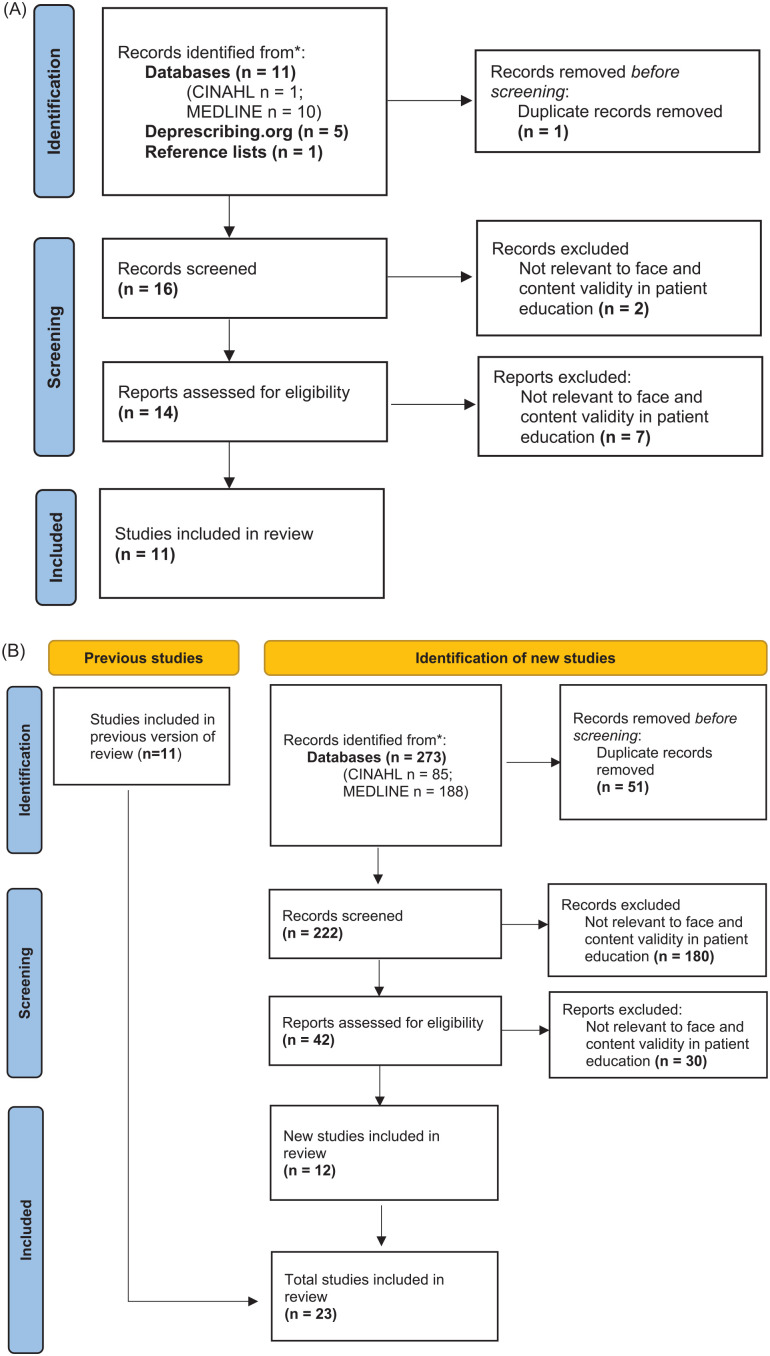
(A) Literature search on patients’ preferences on drug information in patient educational materials. (B) Updated search on patients’ preferences on drug information in patient educational materials.

**Figure 2.**
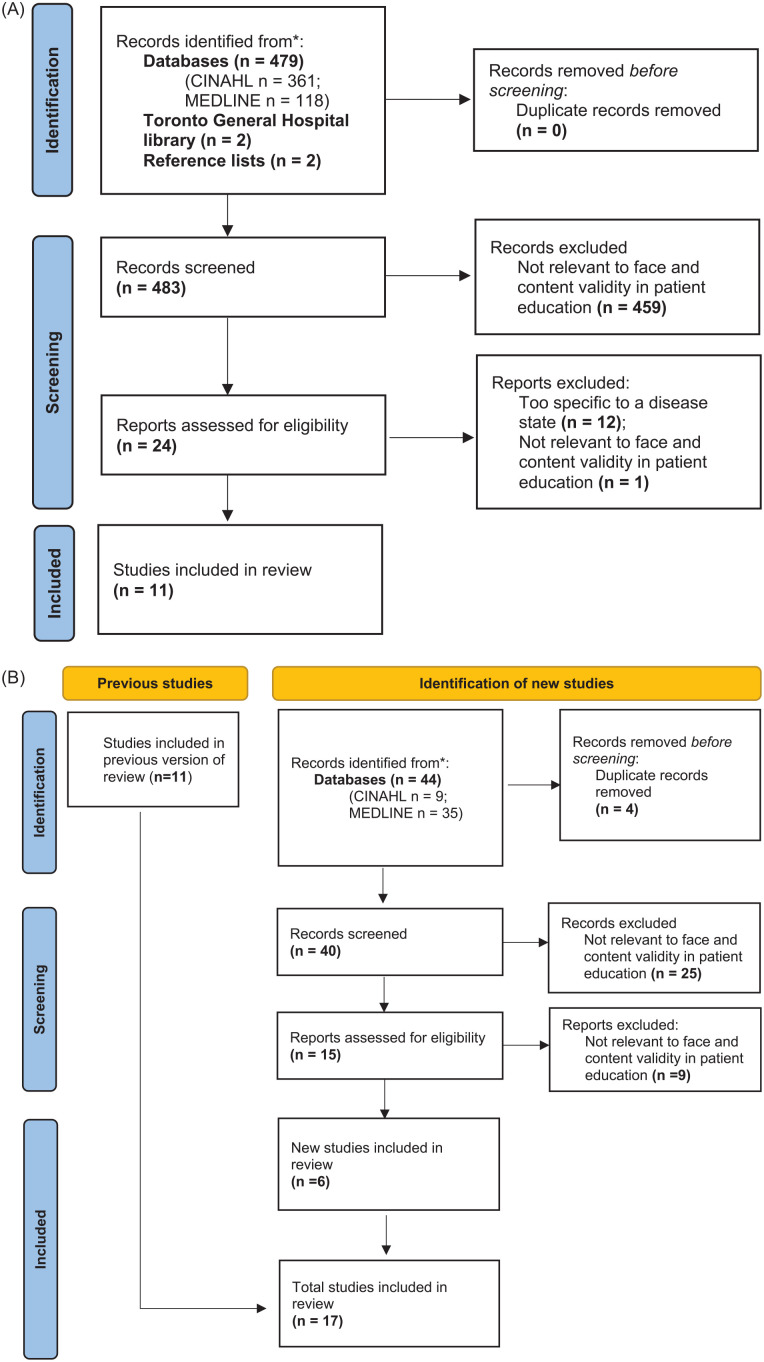
(A) Literature search on existing information on deprescribing in patient education materials. (B) Updated search on existing information on deprescribing in patient education materials.

The September 2017 literature search identified 497 articles, with 22 articles selected as relevant to the study (Figures 1A and 2A). A total of 10 patient education tools (9 medication-specific; 1 about deprescribing) were developed via Lynn’s 3-step development method for content-valid clinical tools. A total of 6 domains were identified in the literature search, and each tool’s contents were structured to match: (1) “What is the drug?” (2) “Why reduce or stop the dose?” (3) “How to safely reduce or stop the drug?” (4) “What will my doctors monitor?” (5) “What will happen if my symptoms change?” and (6) “Other ways to manage my symptoms.”

The initial bulletins were of 1 page, double-sided, and on a letter-sized piece of paper and presented the information from each aforementioned domain in 6 clearly defined sections. Each bulletin was written in a 14-point font, formatted in a single column, displayed both a URL and QR code for its corresponding video, and contained an open section for patients to write their personalized tapering or discontinuation plan. The initial videos followed the bulletin contents word-for-word, using the same information and medication names. Animations and captions were used to make the content easy to follow, while the content overall was presented in a slower speed to accommodate delayed visual and cognitive processing times of elderly patients.^34,35^ Finally, all patient education materials were reviewed by a research librarian at Toronto General Hospital to ensure the use of lay language and appropriately defined medical terms.

#### Supplemental information

Three patient partners from the Can-SOLVE CKD Patient Council were integral in the development and conduct of this project as a part of the Can-SOLVE CKD Network’s emphasis on patient-oriented research. Patient partners provided feedback on format, accessibility, and feasibility of the education tool’s content, validation approach, and supplemental materials. These patient partners were also involved in conducting interviews with patients in Winnipeg and Vancouver.

The patient education toolkits were developed in conjunction with the following items:

1. Deprescribing algorithms: Nationally content-validated medication-specific algorithms including a flow chart and written protocol, intended for a variety of clinical roles in HD.
2. Evidence tables: A summary of key data and studies used to inform algorithm-creation accompanies the clinician’s deprescribing algorithms.
3. Monitoring forms: Single-sheet forms improve the ease of monitoring key patient safety parameters.
4. Prokinetic agents: The tools for these agents were developed and validated using the Lynn method in 2019 as a pilot to the development and validation of all the other medication classes. This tool is also included in Appendix II along with all the other medication deprescribing patient tools.

#### Updated literature search

The updated literature search in August 2022 identified 317 additional articles, with 18 articles selected as relevant to the study (Figures 1B and 2B). These findings reinforced the 6 domains identified from the September 2017 literature search and revealed a growing preference among patients for education materials in multiple media formats, particularly video.

### Phase 2: Content and Face Validation

#### Study participants

Three patient research partners along with 5 clinicians recruited in Toronto conducted an initial validation round. In the period of April 2019 to May 2022, 105 patient participants were recruited from 3 Canadian cities for the subsequent 3 patient validation rounds.

#### Content validation

Overall CVI scores per round for each bulletin and video are shown in Table 1. Per-round CVI scores of the individual components are shown in Appendix I-C and I-D.

**Table 1.** Overall Content Validity Index (CVI) Score of Patient Education Tools, Per Round of Content Validation.

	Round 1 overall		Round 2 overall		Round 3 overall	
	Bulletin	Video	Bulletin	Video	Bulletin	Video
Alpha-1 blockers	0.87	0.87	1.0	1.0	1.0	0.95
Benzodiazepines	0.96	1.0	1.0	1.0	1.0	1.0
Gabapentinoids	0.93	1.0	1.0	1.0	0.98	1.0
Loop diuretics	0.88	0.99	1.0	1.0	1.0	1.0
Prokinetic agents	0.7	1.0	1.0	1.0	1.0	1.0
Proton pump inhibitors	0.92	0.98	1.0	1.0	0.98	1.0
Quinine	1.0	1.0	1.0	0.95	1.0	1.0
Statins	0.98	1.0	0.98	1.0	1.0	1.0
Urate-lowering agents	0.87	0.89	1.0	1.0	1.0	1.0
Deprescribing	0.98	0.95	1.0	0.95	0.96	0.98

In the preliminary clinician validation round, the overall CVI score was 0.95 for bulletins and 0.92 for videos. In patient round 1, overall bulletin CVI scores ranged from 0.70 to 1.0, and overall video CVI scores ranged from 0.87 to 1.0. In patient round 2, overall bulletin CVI scores ranged from 0.98 to 1.0, and overall video CVI scores ranged from 0.95 to 1.0. In patient round 3, overall bulletin CVI scores ranged from 0.96 to 1.0, and overall video CVI scores ranged from 0.95 to 1.0.

#### Face validation

Overall agreement scores across 3 rounds for the 15 face validity statements per bulletin and video are shown in Tables 2 and 3. Agreement scores per bulletin and video for each round are shown in the Appendix I-E and I-F.

**Table 2.** Overall Agreement Face Validity of Patient Education Bulletin (All Rounds).

	Alpha-1 blockers, %	Benzodiazepines, %	Gabapentinoids, %	Loop diuretics, %	Prokinetic agents, %	Proton pump inhibitors, %	Quinine, %	Statins, %	Urate-lowering agents, %	Deprescribing, %
Q1	78	94	94	100	93	94	100	100	83	89
Q2	83	100	100	89	93	100	94	94	72	89
Q3	89	94	100	100	100	94	100	94	89	94
Q4	89	94	100	100	87	94	100	100	89	94
Q5	83	100	100	100	87	100	100	94	83	94
Q6	89	100	100	94	100	100	94	94	94	94
Q7	94	100	100	100	100	94	83	89	89	78
Q8	94	100	100	100	100	100	100	100	94	100
Q9	94	100	100	100	100	100	100	100	89	100
Q10	83	94	94	89	93	94	94	94	78	100
Q11	94	94	94	100	93	100	100	94	94	100
Q12	94	100	100	100	93	100	100	94	94	94
Q13	83	100	100	94	87	94	94	83	83	89
Q14	89	100	100	100	87	100	94	100	89	100
Q15	89	100	100	100	93	100	94	94	89	94

*Note.* Q1 = the patient education sheet is clear and understandable for patients; Q2 = the purpose of the patient education sheet is clear for patients; Q3 = the patient education sheet uses appropriate language and wording for patients; Q4 = health terms and words are appropriately defined; Q5 = it is written at a reading level appropriate for the general public (6^th^-8^th^ grade level); Q6 = the patient education sheet flows in a logical manner; Q7 = examples are used to bridge the gap between what patients know and what is being taught; Q8 = the patient education sheet is appropriately organized into short sections; Q9 = the patient education sheet section titles and subtitles are clear and informative; Q10 = the patient education sheet is visually appealing (eg, color, headings, spacing); Q11 = the font or print size can be easily read by patients; Q12 = only the most important information is given using no more than 3 to 4 main points; Q13 = the visual aids help rather than distract from the content; Q14 = the content is presented in a style that is patient-centered so that the needs of the patient are the most important ones; Q15 = I would be confident using this patient education sheet.

**Table 3.** Overall Agreement Face Validity of Patient Education Video (All Rounds).

	Alpha-1 blockers, %	Benzodiazepines, %	Gabapentinoids, %	Loop diuretics, %	Prokinetic agents, %	Proton pump inhibitors, %	Quinine, %	Statins, %	Urate-lowering agents, %	Deprescribing, %
Q1	83	89	100	100	93	100	100	100	83	100
Q2	89	100	100	100	100	100	94	94	89	100
Q3	89	89	100	100	100	94	94	100	89	100
Q4	89	94	100	100	100	94	100	100	89	100
Q5	89	100	100	100	100	100	94	94	94	100
Q6	78	100	100	100	100	100	100	89	89	89
Q7	94	100	100	94	100	94	100	89	94	100
Q8	94	94	100	94	100	94	94	100	94	100
Q9	94	94	94	100	93	100	83	78	94	89
Q10	89	100	100	100	93	100	94	94	89	100
Q11	89	100	100	100	90	100	100	94	89	94
Q12	89	100	94	100	87	100	94	94	94	100
Q13	83	100	100	94	100	94	100	100	78	94
Q14	89	100	100	100	100	100	94	94	89	94
Q15	94	100	100	94	100	94	100	100	94	94

*Note.* Q1 = the video is clear and understandable for patients; Q2 = the purpose of the video is clear for patients; Q3 = the video uses appropriate language and wording for patients; Q4 = health terms and words are appropriately defined; Q5 = the order of information in the video flows in a logical manner; Q6 = examples are used to bridge the gap between what patients know and what is being taught; Q7 = the video breaks material into short sections; Q8 = the video section titles are informative; Q9 = the video is visually appealing (eg, color, headings, spacing); Q10 = the font or print size can be easily read by patients; Q11 = text on the screen is easy to read; Q12 = the video uses pictures that are clear and uncluttered; Q13 = the voice in the video is clear and easy to hear (eg, not too fast, not garbled); Q14 = the content is presented in a style that is patient-centered so that the needs of the patient are the most important ones; Q15 = I would be confident using this video.

In the preliminary clinician round, agreement ranged from 50% to 100%. In the 3 patient rounds, all bulletins and videos achieved a minimum of 72% overall agreement for the 15 statements, with the majority achieving >90%.

#### Qualitative feedback

A table containing the per-round changes are shown in Appendix I-G. Patients made many suggestions on bulletin formatting related to heading length and section ordering. A larger text size, use of video animations to “draw out” text, and speech clarity were highlighted as strengths. Patients expressed that the pacing of narration and transitions in our videos needed to be slowed further; subsequent interviews did not reveal similar comments after these items were addressed. Patients reacted positively to the utility added by the materials, stating that the content gave them the vocabulary needed to express their thoughts to their health care providers, clarified the prior knowledge of their medications, or that it had enabled them to elaborate on existing knowledge. Overall, patients found that each section of the bulletin and video was presented logically and that each section complimented their learning or knowledge of the medication and deprescribing process.

A major theme was that the patients found many of the medical terms challenging despite a review by the research librarian before patient review. Much of the medical jargon was found to be either unfamiliar or too complex for patients, and many medical terms were replaced with lay alternatives where possible (eg, “swollen legs or feet” for “edema”) or given simplified definitions (ie, “uric acid is responsible for gout attacks”).

The second major theme was related to patients’ unfamiliarity with deprescribing. Initial bulletins contained a section that briefly explained deprescribing; however, patients in the first round stated that the section did not contain enough information. Patients expressed that they were unfamiliar with the concept of deprescribing and its justification, process, risks, and benefits. In response, a dedicated bulletin on deprescribing was generated and then went through 3 rounds of content and face validation.

The final major theme was related to positive feedback on the complimentary nature between the bulletin and video. Patients preferred the video because “the video explained things better and was more straightforward” and that “the diagrams helped with understanding or language barriers.” Some patients also felt that the video augmented their understanding of the content on bulletin, especially when they felt unclear about parts of the bulletin. Overall, participants expressed that the media should be presented together, as having access to both items greatly improved their understanding of the material.

Please see Appendix II for all the patient education tools (bulletin and video) on deprescribing.

## Discussion

To our knowledge, these are the first content-validated, medication-specific, patient education tools on deprescribing for patients on HD. While deprescribing is a potentially safe and effective method for addressing polypharmacy, lack of medication- and disease-specific tools in existing literature is an adoption barrier for both patients and clinicians considering implementing a deprescribing intervention.^18^ In developing tools to address patient-identified knowledge gaps and reduce ambiguity, we feel that these tools can facilitate patient engagement with SDM in the deprescribing process. These tools are not meant to replace discussion or consultation with clinicians but are intended to provide an accessible educational supplement that increases patients’ confidence and promotes health literacy, which should facilitate their ability to engage meaningfully in SDM during the deprescribing process,^24^ thereby increasing patient uptake, long-term sustainability, and likelihood of success of the deprescribing intervention.

Our results show that a diverse set of Canadian patients on HD have judged the content of our tools to be relevant and appropriate for their intended purpose. Despite the lack of a gold standard method for face and content validation, this is the first attempted formal validation of patient education tools for deprescribing in HD. Importantly, this study revealed many insights into the desired content in health education materials and the health literacy levels of patients on HD. Despite an initial review by clinicians and a hospital librarian to ensure lay readability, our interviews showed that there is a notable gap between actual patient health literacy levels and the health care provider’s perception of patient health literacy levels. In this respect, our review process allowed us to identify problematic terminology and substitute it for lay terms or definitions deemed appropriate by patients. Varying levels of health literacy were accommodated for in the design of our tools, such as the inclusion of a video format, as there is literature that suggests that the use of multimodal content can enhance patient comprehension of health information.^36-40^ While we did not explicitly assess this aspect, patients in our study did report that both the video and bulletin were complimentary in understanding the material. Overall, this indicates that our tools can help enable patients’ understanding of the deprescribing process, and this will ultimately help the engagement of patients in the deprescribing process.^41^

Unique to this study was the use of patient partner researchers to conduct interviews in Winnipeg and Vancouver. In addition to institutional privacy training, patient partners received training from the local research assistant in how to interview patients and conduct the study questionnaires. The usage of patient partners as researchers in this context yielded notable differences in the nature of feedback elicited from participants. Researchers elicited more constructive and technical feedback (content and formatting) than the reactive comments (“I didn’t know this”) and experiential narratives (“I have always felt uncomfortable with this medication”) often recorded by our patient partners. We posit that this discrepancy could be caused by the differences in the interview dynamic; patients may have felt more comfortable sharing specific experiences regarding their disease or care received to a patient partner, rather than a research assistant who may be perceived as part of the health care staff.

### Content Validity

Overall CVI scores of each bulletin and video increased with each patient review round. In many cases, the first patient round of bulletin CVI scores were lower than the scores from the preliminary clinician round, suggesting that there are indeed differences in what patients want to know compared with the information provided by clinicians.

### Face Validity

Feedback received in the face validation process reinforced the patient-centered care principles that directed the scope of our study. Patients expressed that a multimodal approach was convenient and conducive to learning about their medications and the deprescribing process; feedback stated that the video could be accessed as needed, while the bulletin served to reinforce the content. Although participants had not explicitly expressed difficulty in communicating with their health care providers, they reacted positively to the inclusion of prompts that encouraged the initiation of these conversations with their providers. A subset of participants also expressed surprise that they could take agency and provide input into their own health care. Many were simply unaware of the risks posed by their own medications and were unfamiliar with the concept of deprescribing in general. Crucially, patients stated that the education materials provided them with the necessary terms and concepts to verbalize their thoughts or clarify opinions that they held but were unable to express. These reactions are consistent with the literature on patients’ willingness to be proactive in their care.^18-26^ Patients may be passive because they do not possess the health literacy levels required to confidently engage with their care provider or because they were simply unaware of the possibility of being an active participant.^18-20^ Educational materials that effectively communicate these health concepts can empower patients to take a more proactive role in the SDM process of their care.^22-24^ In this respect, we feel that the high face validity demonstrated by our education tools can improve patient engagement in a broader patient-centered deprescribing intervention.

### Limitations

Although a formal validation process of patient education materials is described, there are limitations to this study. The validation process involved patients on HD from 3 major Canadian cities; despite an expanded population of interviewees, the findings may not apply to remote or non-Canadian areas. Furthermore, we did not collect demographic data on the patient participants of this study, and therefore, we do not have data on how gender, age, and cultural background may have affected the validation of the education tools. Currently, the tools are only available in English—the Canadian HD population is diverse, and future translations will need to be completed to meet this reality. Furthermore, a validation involving non–English-speaking patients may have revealed cultural differences in the understandings of illness and medication. Patients expressed that the video was an important compliment to the bulletin, but access and ability to use technology can be potential barriers in areas with fewer resources.

## Conclusion

Patient education tools for deprescribing in HD were developed and validated. The information tools are presented in lay language and have high content/face validity because of the extensive patient validation process. These tools will be incorporated in the design of an upcoming project evaluating the implementation of this deprescribing initiative.

## Supplemental Material

sj-docx-1-cjk-10.1177_20543581221150676 – Supplemental material for Development and Validation of Patient Education Tools for Deprescribing in Patients on Hemodialysis Click here for additional data file. Supplemental material, sj-docx-1-cjk-10.1177_20543581221150676 for Development and Validation of Patient Education Tools for Deprescribing in Patients on Hemodialysis by Thomas Hyunwoo Cho, Patrick C. K. Ng, Melissa J. Lefebvre, Arlene Desjarlais, Dennis McCann, Blair Waldvogel, Marcello Tonelli, Amit X. Garg, JoAnne Wilson, Monica Beaulieu, Judith Marin, Cali Orsulak, Melanie Talson, Monica Sharma, Jordanne Feldberg, Clara Bohm and Marisa Battistella in Canadian Journal of Kidney Health and Disease

sj-docx-2-cjk-10.1177_20543581221150676 – Supplemental material for Development and Validation of Patient Education Tools for Deprescribing in Patients on Hemodialysis Click here for additional data file. Supplemental material, sj-docx-2-cjk-10.1177_20543581221150676 for Development and Validation of Patient Education Tools for Deprescribing in Patients on Hemodialysis by Thomas Hyunwoo Cho, Patrick C. K. Ng, Melissa J. Lefebvre, Arlene Desjarlais, Dennis McCann, Blair Waldvogel, Marcello Tonelli, Amit X. Garg, JoAnne Wilson, Monica Beaulieu, Judith Marin, Cali Orsulak, Melanie Talson, Monica Sharma, Jordanne Feldberg, Clara Bohm and Marisa Battistella in Canadian Journal of Kidney Health and Disease
