# Supplementary material for: Development and Validation of Patient Education Tools for Deprescribing in Patients on Hemodialysis
Source: Can J Kidney Health Dis. 2023 Jan 24;10:20543581221150676. doi: 10.1177/20543581221150676 (PMC9880575; doi:10.1177/20543581221150676)
Supplement: sj-docx-1-cjk-10.1177_20543581221150676 – Supplemental material for Development and Validation of Patient Education Tools for Deprescribing in Patients on Hemodialysis [file sj-docx-1-cjk-10.1177_20543581221150676.docx]

Appendix I Contents

[Appendix A: Keywords used in literature searches 2](#_Toc111850908)

[1) Search for patient preferences on drug information in patient education materials 2](#_Toc111850909)

[2) Search for existing information on deprescribing in patient education 2](#_Toc111850910)

[Appendix B: Sample questionnaire for participant validation 3](#_Toc111850911)

[Appendix C: Bulletin Content Validation Index (CVI) scores per medication 7](#_Toc111850912)

[Appendix D: Video Content Validation Index (CVI) scores per medication 9](#_Toc111850913)

[Appendix E: Bulletin level of agreement with face validity statements 11](#_Toc111850914)

[Appendix F: Video level of agreement with face validity statements 13](#_Toc111850915)

[Appendix G: Summary of changes per validation round 15](#_Toc111850916)

[3) Changes made after initial clinician review 15](#_Toc111850917)

[4) Changes made after first patient review round 15](#_Toc111850918)

[5) Changes made after second patient review round 16](#_Toc111850919)

[6) Changes made after the third patient review round 16](#_Toc111850920)

# Appendix A: Keywords used in literature searches

## Search for patient preferences on drug information in patient education materials

***Medline****

Drug

Drug therapy

Patient education as topic

Patient prefer

Patient satisfaction

Patient want

Pharmaceutical preparations

Pharmaceutical services

Prescriptions

***CINAHL****

Drug

Drugs

Drug therapy

Patient education

Patient prefer

Patient satisfaction

Patient want

Pharmacy service

Prescriptions

*All results limited to English language and limited to 1990 to September 1, 2017

## Search for existing information on deprescribing in patient education

***Medline****

Deprescribe

Deprescriptions

Patient education

Patient education as topic

***CINAHL****

Deprescribe

Patient education

*All results limited to English language and limited to 1990 to September 1, 2017

# Appendix B: Sample questionnaire for participant validation

**VALIDATION OF PATIENT INFORMATION SHEET – WHAT IS DEPRESCRIBING?**

Study: Targeted Deprescribing in Hemodialysis Patients to Decrease Polypharmacy

**PART A: CONTENT VALIDITY FOR PATIENT INFORMATION SHEET**

You have been provided with a copy of the patient information sheet. The patient information sheet has been divided into different sections (A, B, C, etc.). Please rate each section on a scale from one (irrelevant) to four (extremely relevant). For any sections you do not find relevant, please provide a comment regarding why. A score of one or two indicates the section should be removed or revised.

1 (Irrelevant) 2 (Less Relevant) 3 (Relevant) 4 (Extremely Relevant)

| SECTION | SCORE | | | | Comments |
| --- | --- | --- | --- | --- | --- |
| A: What is deprescribing? | 1 | 2 | 3 | 4 |  |
| B: Patients on HD take a lot of medications | 1 | 2 | 3 | 4 |  |
| C: Patients on HD may have more side effects from their medications | 1 | 2 | 3 | 4 |  |
| D: What does taking a lot of medicine do? | 1 | 2 | 3 | 4 |  |
| E: Should I deprescribe? | 1 | 2 | 3 | 4 |  |
| F: What if I change my mind? | 1 | 2 | 3 | 4 |  |
| G: Who can I talk to if I have questions/concerns? | 1 | 2 | 3 | 4 |  |
| H: This info is also available in video format | 1 | 2 | 3 | 4 |  |
| I: Contact your healthcare team before stopping any medicines | 1 | 2 | 3 | 4 |  |

1. What important items are missing that should have been included in the patient information sheet?

____________________________________________________________________________________________________________________________________________________________________________________

2. What information is included that should not be included in the patient information sheet?

____________________________________________________________________________________________________________________________________________________________________________________

Additional comments:

____________________________________________________________________________________________________________________________________________________________________________________

**PART B: FACE VALIDATION FOR PATIENT INFORMATION SHEET**

Please circle your level of agreement with each statement in the questionnaire. For any statement which you do not ‘Strongly Agree’ with, please provide a comment regarding why and/or how the patient information sheet can be improved in that aspect. A score of 1, 2, or 3 suggests that the component requires revision.

1 (Strongly Disagree) 2 (Disagree) 3 (Neutral) 4 (Agree) 5 (Strongly Agree)

| **SECTIONS** | **SCORE** | | | | | **COMMENTS** |
| --- | --- | --- | --- | --- | --- | --- |
| The patient information sheet is clear and understandable for patients | 1 | 2 | 3 | 4 | 5 |  |
| The purpose of the patient information sheet is clear for patients | 1 | 2 | 3 | 4 | 5 |  |
| The patient information sheet uses appropriate language and wording for patients | 1 | 2 | 3 | 4 | 5 |  |
| Health terms and words are appropriately defined | 1 | 2 | 3 | 4 | 5 |  |
| It is written at a reading level appropriate for the general public (6-8th grade level) | 1 | 2 | 3 | 4 | 5 |  |
| The patient information sheet flows in a logical manner | 1 | 2 | 3 | 4 | 5 |  |
| Examples are used to bridge the gap between what patients know and what is being taught | 1 | 2 | 3 | 4 | 5 |  |
| The patient information sheet is appropriately organized into short sections | 1 | 2 | 3 | 4 | 5 |  |
| The patient information sheet section titles and subtitles are clear and informative | 1 | 2 | 3 | 4 | 5 |  |
| The patient information sheet is visually appealing (for example: colour, headings, spacing, etc.) | 1 | 2 | 3 | 4 | 5 |  |
| The font or print size can be easily read by patients* | 1 | 2 | 3 | 4 | 5 |  |
| Only the most important information is given using no more than 3 to 4 main points | 1 | 2 | 3 | 4 | 5 |  |
| The visual aids help rather than distract from the content | 1 | 2 | 3 | 4 | 5 |  |
| The content is presented in a style that is patient-centred so that the needs of the patient are most important | 1 | 2 | 3 | 4 | 5 |  |
| I would be confident using this patient information sheet | 1 | 2 | 3 | 4 | 5 |  |

* 14-point font recommended for the elderly; 12-point font recommended for general audiences

1. Is there information on this patient information sheet that would be problematic for patients? YES / NO

What information on this patient information sheet would be problematic for patients?

____________________________________________________________________________________________________________________________________________________________________________________

2. What wording changes, if any, would you recommend to improve understandability for patients?

____________________________________________________________________________________________________________________________________________________________________________________

3. Additional Comments:

____________________________________________________________________________________________________________________________________________________________________________________

**VALIDATION OF PATIENT INFORMATION VIDEO – WHAT IS DEPRESCRIBING?**

Study: Targeted Deprescribing in Hemodialysis Patients to Decrease Polypharmacy

**PART A: CONTENT VALIDITY FOR PATIENT INFORMATION VIDEO**

You have been provided with a copy of the patient information video. The patient information video has been divided into different sections (A, B, C, etc.). Please rate each section on a scale from one (irrelevant) to four (extremely relevant). For any sections you do not find relevant, please provide a comment regarding why. A score of one or two indicates the section should be removed or revised.

1 (Irrelevant) 2 (Less Relevant) 3 (Relevant) 4 (Extremely Relevant)

| SECTION | SCORE | | | | Comments |
| --- | --- | --- | --- | --- | --- |
| A: What is deprescribing? | 1 | 2 | 3 | 4 |  |
| B: Patients on HD take a lot of medications | 1 | 2 | 3 | 4 |  |
| C: Patients on HD may have more side effects from their medications | 1 | 2 | 3 | 4 |  |
| D: What does taking a lot of medicine do? | 1 | 2 | 3 | 4 |  |
| E: Should I deprescribe? | 1 | 2 | 3 | 4 |  |
| F: What if I change my mind? | 1 | 2 | 3 | 4 |  |
| G: Who can I talk to if I have questions/concerns? | 1 | 2 | 3 | 4 |  |
| H: This info is also available in video format | 1 | 2 | 3 | 4 |  |
| I: Contact your healthcare team before stopping any medicines | 1 | 2 | 3 | 4 |  |

1. What important items are missing that should have been included in the patient information video?
____________________________________________________________________________________________________________________________________________________________________________________

2. What information is included that should not be included in the patient information video?

____________________________________________________________________________________________________________________________________________________________________________________

Additional comments:

____________________________________________________________________________________________________________________________________________________________________________________

**PART B: FACE VALIDATION FOR PATIENT INFORMATION VIDEO**

Please circle your level of agreement with each statement in the questionnaire. For any statement which you do not ‘Strongly Agree’ with, please provide a comment regarding why and/or how the patient information video can be improved in that aspect. A score of 1, 2, or 3 for any statement suggests that the component requires revision.

1 (Strongly Disagree) 2 (Disagree) 3 (Neutral) 4 (Agree) 5 (Strongly Agree)

| **SECTIONS** | **SCORE** | | | | | **COMMENTS** |
| --- | --- | --- | --- | --- | --- | --- |
| The information in the video is easily understood by patients | 1 | 2 | 3 | 4 | 5 |  |
| The purpose of the patient information video is clear for patients | 1 | 2 | 3 | 4 | 5 |  |
| The video uses appropriate language and words for patients | 1 | 2 | 3 | 4 | 5 |  |
| Health terms and words are appropriately defined | 1 | 2 | 3 | 4 | 5 |  |
| The order of information in the video flows in a logical manner | 1 | 2 | 3 | 4 | 5 |  |
| Examples are used to bridge the gap between what patients know and what is being taught | 1 | 2 | 3 | 4 | 5 |  |
| The video breaks material into short sections | 1 | 2 | 3 | 4 | 5 |  |
| The video section titles are informative | 1 | 2 | 3 | 4 | 5 |  |
| The video is visually appealing (for example: colour, headings, spacing, etc.) | 1 | 2 | 3 | 4 | 5 |  |
| Font or print size can be easily read by patients * | 1 | 2 | 3 | 4 | 5 |  |
| Text on the screen is easy to read | 1 | 2 | 3 | 4 | 5 |  |
| The video uses pictures that are clear and uncluttered | 1 | 2 | 3 | 4 | 5 |  |
| The voice in the video is clear and easy to hear (for example: not too fast, not garbled) | 1 | 2 | 3 | 4 | 5 |  |
| The content is presented in a style that is patient-centred so that the needs of the patient are most important | 1 | 2 | 3 | 4 | 5 |  |
| I would be confident using this patient information video | 1 | 2 | 3 | 4 | 5 |  |

* 14-point font recommended for the elderly; 12-point font recommended for general audiences

1. Is there any information in this video that would be problematic for patients? YES / NO

What information on this patient information video would be problematic for patients?

____________________________________________________________________________________________________________________________________________________________________________________

2. What wording changes, if any, would you recommend to improve understandability for patients?

____________________________________________________________________________________________________________________________________________________________________________________

3. Did the sheet and video help with fears about deprescribing? Circle: YES / NO

4. Did the sheet and video increase your knowledge about deprescribing? YES / NO

Additional Comments:

____________________________________________________________________________________________________________________________________________________________________________________

# Appendix C: Bulletin Content Validation Index (CVI) scores per medication

| **Alpha-1-Blockers** | | | | | | | | | | | | |
| --- | --- | --- | --- | --- | --- | --- | --- | --- | --- | --- | --- | --- |
| **Bulletin Component** | **A** | **B** | **C** | **D** | **E** | **F** | **G** | **H** | **I** | **J** |  | **Mean CVI** |
| **Clinician Round** | 1.0 | 1.0 | 1.0 | 1.0 | 1.0 | 1.0 | 1.0 | 1.0 | 1.0 | 1.0 |  | 1.0 |
| **Round 1** | 0.67 | 0.83 | 0.83 | 0.83 | 1.0 | 1.0 | 0.83 | 0.83 | 0.83 | 1.0 |  | 0.87 |
| **Round 2** | 1.0 | 1.0 | 1.0 | 1.0 | 1.0 | 1.0 | 1.0 | 1.0 | 1.0 | N/A |  | 1.0 |
| **Round 3** | 1.0 | 1.0 | 1.0 | 1.0 | 1.0 | 1.0 | 1.0 | 1.0 | 1.0 | N/A |  | 1.0 |
| **Benzodiazepines / Z-Drugs** | | | | | | | | | | | | |
| **Bulletin Component** | **A** | **B** | **C** | **D** | **E** | **F** | **G** | **H** | **I** |  |  | **Mean CVI** |
| **Clinician Round** | 1.0 | 1.0 | 1.0 | 1.0 | 1.0 | 1.0 | 0.75 | 1.0 | 1.0 |  |  | 1.0 |
| **Round 1** | 1.0 | 1.0 | 0.83 | 1.0 | 1.0 | 1.0 | 1.0 | 1.0 | 0.83 |  |  | 0.96 |
| **Round 2** | 1.0 | 1.0 | 1.0 | 1.0 | 1.0 | 1.0 | 1.0 | 1.0 | N/A |  |  | 1.0 |
| **Round 3** | 1.0 | 1.0 | 1.0 | 1.0 | 1.0 | 1.0 | 1.0 | 1.0 | N/A |  |  | 1.0 |
| **Gabapentinoids** | | | | | | | | | | | | |
| **Bulletin Component** | **A** | **B** | **C** | **D** | **E** | **F** | **G** | **H** | **I** |  |  | **Mean CVI** |
| **Clinician Round** | 1.0 | 1.0 | 1.0 | 1.0 | 1.0 | 0.8 | 1.0 | 1.0 | 1.0 |  |  | 1.0 |
| **Round 1** | 1.0 | 1.0 | 0.83 | 0.83 | 0.83 | 1.0 | 1.0 | 0.83 | 1.0 |  |  | 0.93 |
| **Round 2** | 1.0 | 1.0 | 1.0 | 1.0 | 1.0 | 1.0 | 1.0 | 1.0 | N/A |  |  | 1.0 |
| **Round 3** | 1.0 | 0.83 | 1.0 | 1.0 | 1.0 | 1.0 | 1.0 | 1.0 | N/A |  |  | 0.98 |
| **Loop Diuretics** | | | | | | | | | | | | |
| **Bulletin Component** | **A** | **B** | **C** | **D** | **E** | **F** | **G** | **H** | **I** | **J** | **K** | **Mean CVI** |
| **Clinician Round** | 1.0 | 1.0 | 0.8 | 1.0 | 1.0 | 1.0 | 0.8 | 0.8 | 1.0 | 1.0 | 1.0 | 1.0 |
| **Round 1** | 0.83 | 0.67 | 0.67 | 0.83 | 0.83 | 1.0 | 1.0 | 1.0 | 1.0 | 1.0 | N/A | 0.88 |
| **Round 2** | 1.0 | 1.0 | 1.0 | 1.0 | 1.0 | 1.0 | 1.0 | 1.0 | 1.0 | N/A | N/A | 1.0 |
| **Round 3** | 1.0 | 1.0 | 1.0 | 1.0 | 1.0 | 1.0 | 1.0 | 1.0 | 1.0 | N/A | N/A | 1.0 |
| **Prokinetic Agents** | | | | | | | | | | | | |
| **Bulletin Component** | **A** | **B** | **C** | **D** | **E** | **F** | **G** | **H** | **I** | **J** | **K** | **Mean CVI** |
| **Clinician Round** | 1.0 | 1.0 | 1.0 | 1.0 | 1.0 | 1.0 | 1.0 | 1.0 | 1.0 | 1.0 | N/A | 1.0 |
| **Round 1** | 1.0 | 1.0 | 0.4 | 1.0 | 1.0 | 0.8 | 1.0 | 0.6 | 1.0 | 1.0 | N/A | 0.7 |
| **Round 2** | 1.0 | 1.0 | 1.0 | 1.0 | 1.0 | 1.0 | 1.0 | 1.0 | 1.0 | 1.0 | N/A | 1.0 |
| **Round 3** | 1.0 | 1.0 | 1.0 | 1.0 | 1.0 | 1.0 | 1.0 | 1.0 | 1.0 | 1.0 | N/A | 1.0 |
| **Proton Pump Inhibitors** | | | | | | | | | | | | |
| **Bulletin Component** | **A** | **B** | **C** | **D** | **E** | **F** | **G** | **H** | **I** | **J** | **K** | **Mean CVI** |
| **Clinician Round** | 1.0 | 1.0 | 0.8 | 1.0 | 0.8 | 1.0 | 1.0 | 1.0 | 1.0 | 1.0 | 1.0 | 0.96 |
| **Round 1** | 0.83 | 0.83 | 0.83 | 0.83 | 1.0 | 0.83 | 1.0 | 1.0 | 1.0 | 1.0 | 1.0 | 0.92 |
| **Round 2** | 1.0 | 1.0 | 1.0 | 1.0 | 1.0 | 1.0 | 1.0 | 1.0 | 1.0 | 1.0 | N/A | 1.0 |
| **Round 3** | 0.83 | 1.0 | 1.0 | 1.0 | 1.0 | 1.0 | 1.0 | 1.0 | 1.0 | 1.0 | N/A | 0.98 |
| **Quinines** | | | | | | | | | | | | |
| **Bulletin Component** | **A** | **B** | **C** | **D** | **E** | **F** | **G** | **H** | **I** | **J** |  | **Mean CVI** |
| **Clinician Round** | 1.0 | 0.8 | 1.0 | 0.6 | 0.6 | 0.6 | 0.4 | 0.4 | 0.8 | 1.0 |  | 0.72 |
| **Round 1** | 1.0 | 1.0 | 1.0 | 1.0 | 1.0 | 1.0 | 1.0 | 1.0 | 1.0 | N/A |  | 1.0 |
| **Round 2** | 1.0 | 1.0 | 1.0 | 1.0 | 1.0 | 1.0 | 1.0 | 1.0 | 1.0 | N/A |  | 1.0 |
| **Round 3** | 1.0 | 1.0 | 1.0 | 1.0 | 1.0 | 1.0 | 1.0 | 1.0 | 1.0 | N/A |  | 1.0 |
| **Statins** | | | | | | | | | | | | |
| **Bulletin Component** | **A** | **B** | **C** | **D** | **E** | **F** | **G** | **H** | **I** |  |  | **Mean CVI** |
| **Clinician Round** | 1.0 | 0.8 | 1.0 | 1.0 | 0.6 | 0.8 | 0.8 | 1.0 | 1.0 |  |  | 0.89 |
| **Round 1** | 1.0 | 1.0 | 1.0 | 1.0 | 1.0 | 1.0 | 0.83 | 1.0 | 1.0 |  |  | 0.98 |
| **Round 2** | 1.0 | 1.0 | 1.0 | 1.0 | 1.0 | 1.0 | 0.83 | 1.0 | N/A |  |  | 0.98 |
| **Round 3** | 1.0 | 1.0 | 1.0 | 1.0 | 1.0 | 1.0 | 1.0 | 1.0 | N/A |  |  | 1.0 |
| **Urate Lowering Agents** | | | | | | | | | | | | |
| **Bulletin Component** | **A** | **B** | **C** | **D** | **E** | **F** | **G** | **H** | **I** | **J** |  | **Mean CVI** |
| **Clinician Round** | 1.0 | 1.0 | 1.0 | 1.0 | 1.0 | 0.8 | 1.0 | 1.0 | 1.0 | 1.0 |  | 0.98 |
| **Round 1** | 0.67 | 0.83 | 0.83 | 0.83 | 1.0 | 1.0 | 0.83 | 0.83 | 0.83 | 1.0 |  | 0.87 |
| **Round 2** | 1.0 | 1.0 | 1.0 | 1.0 | 1.0 | 1.0 | 1.0 | 1.0 | 1.0 | N/A |  | 1.0 |
| **Round 3** | 1.0 | 1.0 | 1.0 | 1.0 | 1.0 | 1.0 | 1.0 | 1.0 | 1.0 | N/A |  | 1.0 |
| **General Deprescribing** | | | | | | | | | | | | |
| **Bulletin Component** | **A** | **B** | **C** | **D** | **E** | **F** | **G** | **H** | **I** |  |  | **Mean CVI** |
| **Round 1** | 1.0 | 0.83 | 1.0 | 1.0 | 1.0 | 1.0 | 1.0 | 1.0 | 1.0 |  |  | 0.98 |
| **Round 2** | 1.0 | 1.0 | 1.0 | 1.0 | 1.0 | 1.0 | 1.0 | 1.0 | 1.0 |  |  | 1.0 |
| **Round 3** | 1.0 | 1.0 | 0.83 | 1.0 | 1.0 | 1.0 | 1.0 | 0.83 | 1.0 |  |  | 0.96 |

# Appendix D: Video Content Validation Index (CVI) scores per medication

| **Alpha-1-Blockers** | | | | | | | | | | | | |
| --- | --- | --- | --- | --- | --- | --- | --- | --- | --- | --- | --- | --- |
| **Video Component** | **A** | **B** | **C** | **D** | **E** | **F** | **G** | **H** | **I** |  |  | **Mean CVI** |
| **Clinician Round** | 1.0 | 1.0 | 1.0 | 1.0 | 1.0 | 1.0 | 1.0 | 0.8 | 1.0 |  |  | 0.98 |
| **Round 1** | 0.83 | 0.83 | 0.67 | 0.67 | 1.0 | 1.0 | 1.0 | 0.83 | 1.0 |  |  | 0.87 |
| **Round 2** | 1.0 | 1.0 | 1.0 | 1.0 | 1.0 | 1.0 | 1.0 | N/A | N/A |  |  | 1.0 |
| **Round 3** | 1.0 | 1.0 | 1.0 | 1.0 | 0.83 | 0.83 | 1.0 | N/A | N/A |  |  | 0.95 |
| **Benzodiazepines / Z-Drugs** | | | | | | | | | | | | |
| **Video Component** | **A** | **B** | **C** | **D** | **E** | **F** | **G** | **H** | **I** |  |  | **Mean CVI** |
| **Clinician Round** | 1.0 | 1.0 | 1.0 | 1.0 | 1.0 | 1.0 | 1.0 | 1.0 | 1.0 |  |  | 1.0 |
| **Round 1** | 1.0 | 1.0 | 1.0 | 1.0 | 1.0 | 1.0 | 1.0 | 1.0 | 1.0 |  |  | 1.0 |
| **Round 2** | 1.0 | 1.0 | 1.0 | 1.0 | 1.0 | 1.0 | 1.0 | N/A | N/A |  |  | 1.0 |
| **Round 3** | 1.0 | 1.0 | 1.0 | 1.0 | 1.0 | 1.0 | 1.0 | N/A | N/A |  |  | 1.0 |
| **Gabapentinoids** | | | | | | | | | | | | |
| **Video Component** | **A** | **B** | **C** | **D** | **E** | **F** | **G** | **H** | **I** |  |  | **Mean CVI** |
| **Clinician Round** | 1.0 | 1.0 | 1.0 | 1.0 | 1.0 | 0.8 | 0.8 | 1.0 | 1.0 |  |  | 0.96 |
| **Round 1** | 1.0 | 1.0 | 1.0 | 1.0 | 1.0 | 1.0 | 1.0 | 1.0 | 1.0 |  |  | 1.0 |
| **Round 2** | 1.0 | 1.0 | 1.0 | 1.0 | 1.0 | 1.0 | 1.0 | N/A | N/A |  |  | 1.0 |
| **Round 3** | 1.0 | 1.0 | 1.0 | 1.0 | 1.0 | 1.0 | 1.0 | N/A | N/A |  |  | 1.0 |
| **Loop Diuretics** | | | | | | | | | | | | |
| **Video Component** | **A** | **B** | **C** | **D** | **E** | **F** | **G** | **H** | **I** | **J** | **K** | **Mean CVI** |
| **Clinician Round** | 1.0 | 1.0 | 1.0 | 1.0 | 1.0 | 1.0 | 0.8 | 1.0 | 1.0 | N/A | N/A | 0.98 |
| **Round 1** | 1.0 | 1.0 | 0.83 | 1.0 | 1.0 | 1.0 | 1.0 | 1.0 | 1.0 | 1.0 | 1.0 | 0.99 |
| **Round 2** | 1.0 | 1.0 | 1.0 | 1.0 | 1.0 | 1.0 | 1.0 | N/A | N/A | N/A | N/A | 1.0 |
| **Round 3** | 1.0 | 1.0 | 1.0 | 1.0 | 1.0 | 1.0 | 1.0 | N/A | N/A | N/A | N/A | 1.0 |
| **Prokinetic Agents** | | | | | | | | | | | | |
| **Video Component** | **A** | **B** | **C** | **D** | **E** | **F** | **G** | **H** | **I** | **J** | **K** | **Mean CVI** |
| **Clinician Round** | 1.0 | 1.0 | 1.0 | 1.0 | 1.0 | 1.0 | 1.0 | 1.0 | 1.0 | 1.0 | N/A | 1.0 |
| **Round 1** | 1.0 | 1.0 | 1.0 | 1.0 | 1.0 | 1.0 | 1.0 | 1.0 | 1.0 | 1.0 | N/A | 1.0 |
| **Round 2** | 1.0 | 1.0 | 1.0 | 1.0 | 1.0 | 1.0 | 1.0 | 1.0 | 1.0 | 1.0 | N/A | 1.0 |
| **Round 3** | 1.0 | 1.0 | 1.0 | 1.0 | 1.0 | 1.0 | 1.0 | 1.0 | 1.0 | 1.0 | N/A | 1.0 |
| **Proton Pump Inhibitors** | | | | | | | | | | | | |
| **Video Component** | **A** | **B** | **C** | **D** | **E** | **F** | **G** | **H** | **I** | **J** |  | **Mean CVI** |
| **Clinician Round** | 1.0 | 1.0 | 0.8 | 1.0 | 0.8 | 1.0 | 1.0 | 1.0 | 1.0 | 1.0 |  | 0.96 |
| **Round 1** | 1.0 | 1.0 | 0.83 | 1.0 | 1.0 | 1.0 | 1.0 | 1.0 | 1.0 | 1.0 |  | 0.98 |
| **Round 2** | 1.0 | 1.0 | 1.0 | 1.0 | 1.0 | 1.0 | 1.0 | 1.0 | N/A | N/A |  | 1.0 |
| **Round 3** | 1.0 | 1.0 | 1.0 | 1.0 | 1.0 | 1.0 | 1.0 | 1.0 | N/A | N/A |  | 1.0 |
| **Quinines** | | | | | | | | | | | | |
| **Video Component** | **A** | **B** | **C** | **D** | **E** | **F** | **G** | **H** | **I** |  |  | **Mean CVI** |
| **Clinician Round** | 1.0 | 0.75 | 1.0 | 0.5 | 0.5 | 0.5 | 0.25 | 0.25 | 1.0 |  |  | 0.64 |
| **Round 1** | 1.0 | 1.0 | 1.0 | 1.0 | 1.0 | 1.0 | 1.0 | N/A | N/A |  |  | 1.0 |
| **Round 2** | 0.83 | 1.0 | 1.0 | 1.0 | 1.0 | 1.0 | 0.83 | N/A | N/A |  |  | 0.95 |
| **Round 3** | 1.0 | 1.0 | 1.0 | 1.0 | 1.0 | 1.0 | 1.0 | N/A | N/A |  |  | 1.0 |
| **Statins** | | | | | | | | | | | | |
| **Video Component** | **A** | **B** | **C** | **D** | **E** | **F** | **G** | **H** |  |  |  | **Mean CVI** |
| **Clinician Round** | 1.0 | 0.8 | 1.0 | 1.0 | 0.8 | 0.6 | 0.75 | 1.0 |  |  |  | 0.87 |
| **Round 1** | 1.0 | 1.0 | 1.0 | 1.0 | 1.0 | 1.0 | 1.0 | 1.0 |  |  |  | 1.0 |
| **Round 2** | 1.0 | 1.0 | 1.0 | 1.0 | 1.0 | 1.0 | N/A | N/A |  |  |  | 1.0 |
| **Round 3** | 1.0 | 1.0 | 1.0 | 1.0 | 1.0 | 1.0 | N/A | N/A |  |  |  | 1.0 |
| **Urate Lowering Agents** | | | | | | | | | | | | |
| **Video Component** | **A** | **B** | **C** | **D** | **E** | **F** | **G** | **H** | **I** |  |  | **Mean CVI** |
| **Clinician Round** | 1.0 | 1.0 | 1.0 | 1.0 | 1.0 | 0.8 | 0.8 | 1.0 | 1.0 |  |  | 0.96 |
| **Round 1** | 0.83 | 0.83 | 0.67 | 0.83 | 1.0 | 1.0 | 1.0 | 0.83 | 1.0 |  |  | 0.89 |
| **Round 2** | 1.0 | 1.0 | 1.0 | 1.0 | 1.0 | 1.0 | 1.0 | N/A | N/A |  |  | 1.0 |
| **Round 3** | 1.0 | 1.0 | 1.0 | 1.0 | 1.0 | 1.0 | 1.0 | N/A | N/A |  |  | 1.0 |
| **General Deprescribing** | | | | | | | | | | | | |
| **Video Component** | **A** | **B** | **C** | **D** | **E** | **F** | **G** | **H** | **I** |  |  | **Mean CVI** |
| **Round 1** | 1.0 | 0.83 | 1.0 | 1.0 | 1.0 | 1.0 | 0.83 | 1.0 | 1.0 |  |  | 0.95 |
| **Round 2** | 1.0 | 1.0 | 0.83 | 1.0 | 1.0 | 1.0 | 0.83 | 1.0 | 1.0 |  |  | 0.95 |
| **Round 3** | 1.0 | 1.0 | 0.83 | 1.0 | 1.0 | 1.0 | 1.0 | 1.0 | 1.0 |  |  | 0.98 |

# Appendix E: Bulletin level of agreement with face validity statements

**Face validity statements:**

1. The patient information sheet is clear and understandable for patients
2. The purpose of the patient information sheet is clear for patients
3. The patient information sheet uses appropriate language and wording for patients
4. Health terms and words are appropriately defined
5. It is written at a reading level appropriate for the general public (6-8th grade)
6. The patient information sheet flows in a logical manner
7. Examples are used to bridge the gap between what patients know and what is being taught
8. The patient information sheet is appropriately organized into short sections
9. The patient information sheet section titles and subtitles are clear and informative
10. The patient information sheet is visually appealing (example: colour, heading, spacing)
11. The font or print size can be easily read by patients
12. Only the most important information is given, using no more than 3 to 4 main points
13. The visual aids help rather than distract from the content
14. The content is presented in a style that is patient-centred so that the needs of the patients are most important
15. I would be confident using this patient information sheet

| **Overall levels of agreement per medication** | | | | | | | | | | | | | | | |
| --- | --- | --- | --- | --- | --- | --- | --- | --- | --- | --- | --- | --- | --- | --- | --- |
| **Alpha-1-Blockers** | | | | | | | | | | | | | | | |
| **Statement** | **1** | **2** | **3** | **4** | **5** | **6** | **7** | **8** | **9** | **10** | **11** | **12** | **13** | **14** | **15** |
| **Clinician Round** | 100% | 100% | 100% | 100% | 100% | 100% | 100% | 100% | 100% | 80% | 100% | 100% | 100% | 100% | 100% |
| **Round 1** | 50% | 67% | 83% | 83% | 67% | 67% | 83% | 83% | 83% | 67% | 83% | 83% | 67% | 67% | 67% |
| **Round 2** | 100% | 100% | 100% | 100% | 100% | 100% | 100% | 100% | 100% | 100% | 100% | 100% | 100% | 100% | 100% |
| **Round 3** | 83% | 83% | 83% | 83% | 83% | 100% | 100% | 100% | 100% | 83% | 100% | 100% | 83% | 100% | 100% |
| **Benzodiazepines / Z-Drugs** | | | | | | | | | | | | | | | |
| **Statement** | **1** | **2** | **3** | **4** | **5** | **6** | **7** | **8** | **9** | **10** | **11** | **12** | **13** | **14** | **15** |
| **Clinician Round** | 100% | 100% | 100% | 100% | 75% | 100% | 75% | 75% | 100% | 75% | 100% | 75% | 75% | 100% | 100% |
| **Round 1** | 100% | 100% | 100% | 100% | 100% | 100% | 100% | 100% | 100% | 83% | 83% | 100% | 100% | 100% | 100% |
| **Round 2** | 100% | 100% | 100% | 100% | 100% | 100% | 100% | 100% | 100% | 100% | 100% | 100% | 100% | 100% | 100% |
| **Round 3** | 83% | 100% | 83% | 83% | 100% | 100% | 100% | 100% | 100% | 100% | 100% | 100% | 100% | 100% | 100% |
| **Gabapentinoids** | | | | | | | | | | | | | | | |
| **Statement** | **1** | **2** | **3** | **4** | **5** | **6** | **7** | **8** | **9** | **10** | **11** | **12** | **13** | **14** | **15** |
| **Clinician Round** | 100% | 100% | 100% | 100% | 80% | 100% | 80% | 100% | 100% | 80% | 100% | 80% | 60% | 100% | 100% |
| **Round 1** | 83% | 100% | 100% | 100% | 100% | 100% | 100% | 100% | 100% | 83% | 83% | 100% | 100% | 100% | 100% |
| **Round 2** | 100% | 100% | 100% | 100% | 100% | 100% | 100% | 100% | 100% | 100% | 100% | 100% | 100% | 100% | 100% |
| **Round 3** | 100% | 100% | 100% | 100% | 100% | 100% | 100% | 100% | 100% | 100% | 100% | 100% | 100% | 100% | 100% |
| **Loop Diuretics** | | | | | | | | | | | | | | | |
| **Statement** | **1** | **2** | **3** | **4** | **5** | **6** | **7** | **8** | **9** | **10** | **11** | **12** | **13** | **14** | **15** |
| **Clinician Round** | 100% | 75% | 100% | 100% | 100% | 100% | 100% | 100% | 100% | 80% | 60% | 100% | 60% | 100% | 80% |
| **Round 1** | 100% | 100% | 100% | 100% | 100% | 83% | 100% | 100% | 100% | 67% | 100% | 100% | 100% | 100% | 100% |
| **Round 2** | 100% | 100% | 100% | 100% | 100% | 100% | 100% | 100% | 100% | 100% | 100% | 100% | 100% | 100% | 100% |
| **Round 3** | 100% | 67% | 100% | 100% | 100% | 100% | 100% | 100% | 100% | 100% | 100% | 100% | 83% | 100% | 100% |
| **Prokinetic Agents** | | | | | | | | | | | | | | | |
| **Statement** | **1** | **2** | **3** | **4** | **5** | **6** | **7** | **8** | **9** | **10** | **11** | **12** | **13** | **14** | **15** |
| **Clinician Round** | 80% | 100% | 80% | 60% | 80% | 100% | 80% | 100% | 100% | 100% | 100% | 80% | 100% | 80% | 80% |
| **Round 1** | 80% | 80% | 100% | 60% | 60% | 100% | 100% | 100% | 100% | 80% | 100% | 100% | 80% | 80% | 100% |
| **Round 2** | 100% | 100% | 100% | 100% | 100% | 100% | 100% | 100% | 100% | 100% | 80% | 80% | 80% | 80% | 80% |
| **Round 3** | 100% | 100% | 100% | 100% | 100% | 100% | 100% | 100% | 100% | 100% | 100% | 100% | 100% | 100% | 100% |
| **Proton Pump Inhibitors** | | | | | | | | | | | | | | | |
| **Statement** | **1** | **2** | **3** | **4** | **5** | **6** | **7** | **8** | **9** | **10** | **11** | **12** | **13** | **14** | **15** |
| **Clinician Round** | 80% | 100% | 100% | 80% | 80% | 100% | 100% | 100% | 100% | 80% | 60% | 80% | 20% | 80% | 80% |
| **Round 1** | 83% | 100% | 100% | 100% | 100% | 100% | 83% | 100% | 100% | 83% | 100% | 100% | 83% | 100% | 100% |
| **Round 2** | 100% | 100% | 100% | 100% | 100% | 100% | 100% | 100% | 100% | 100% | 100% | 100% | 100% | 100% | 100% |
| **Round 3** | 100% | 100% | 83% | 83% | 100% | 100% | 100% | 100% | 100% | 100% | 100% | 100% | 100% | 100% | 100% |
| **Quinines** | | | | | | | | | | | | | | | |
| **Statement** | **1** | **2** | **3** | **4** | **5** | **6** | **7** | **8** | **9** | **10** | **11** | **12** | **13** | **14** | **15** |
| **Clinician Round** | 100% | 100% | 100% | 60% | 100% | 60% | 100% | 100% | 100% | 60% | 80% | 60% | 80% | 100% | 100% |
| **Round 1** | 100% | 100% | 100% | 100% | 100% | 100% | 100% | 100% | 100% | 100% | 100% | 100% | 100% | 100% | 100% |
| **Round 2** | 100% | 83% | 100% | 100% | 100% | 83% | 50% | 100% | 100% | 83% | 100% | 100% | 83% | 83% | 83% |
| **Round 3** | 100% | 100% | 100% | 100% | 100% | 100% | 100% | 100% | 100% | 100% | 100% | 100% | 100% | 100% | 100% |
| **Statins** | | | | | | | | | | | | | | | |
| **Statement** | **1** | **2** | **3** | **4** | **5** | **6** | **7** | **8** | **9** | **10** | **11** | **12** | **13** | **14** | **15** |
| **Clinician Round** | 100% | 100% | 100% | 100% | 100% | 100% | 100% | 100% | 100% | 80% | 80% | 100% | 80% | 100% | 80% |
| **Round 1** | 100% | 100% | 83% | 100% | 100% | 100% | 100% | 100% | 100% | 100% | 100% | 100% | 83% | 100% | 100% |
| **Round 2** | 100% | 83% | 100% | 100% | 83% | 83% | 67% | 100% | 100% | 83% | 83% | 83% | 67% | 100% | 83% |
| **Round 3** | 100% | 100% | 100% | 100% | 100% | 100% | 100% | 100% | 100% | 100% | 100% | 100% | 100% | 100% | 100% |
| **Urate Lowering Agents** | | | | | | | | | | | | | | | |
| **Statement** | **1** | **2** | **3** | **4** | **5** | **6** | **7** | **8** | **9** | **10** | **11** | **12** | **13** | **14** | **15** |
| **Clinician Round** | 100% | 100% | 80% | 80% | 80% | 100% | 100% | 100% | 100% | 80% | 100% | 100% | 100% | 100% | 100% |
| **Round 1** | 50% | 67% | 83% | 83% | 67% | 83% | 67% | 83% | 67% | 67% | 83% | 83% | 50% | 67% | 67% |
| **Round 2** | 100% | 83% | 100% | 100% | 100% | 100% | 100% | 100% | 100% | 83% | 100% | 100% | 100% | 100% | 100% |
| **Round 3** | 100% | 67% | 83% | 83% | 83% | 100% | 100% | 100% | 100% | 83% | 100% | 100% | 100% | 100% | 100% |
| **General Deprescribing** | | | | | | | | | | | | | | | |
| **Statement** | **1** | **2** | **3** | **4** | **5** | **6** | **7** | **8** | **9** | **10** | **11** | **12** | **13** | **14** | **15** |
| **Round 1** | 100% | 100% | 100% | 100% | 100% | 100% | 67% | 100% | 100% | 100% | 100% | 100% | 100% | 100% | 100% |
| **Round 2** | 83% | 83% | 100% | 83% | 83% | 83% | 83% | 100% | 100% | 100% | 100% | 83% | 83% | 100% | 83% |
| **Round 3** | 83% | 83% | 83% | 100% | 100% | 100% | 83% | 100% | 100% | 100% | 100% | 100% | 83% | 100% | 100% |

# Appendix F: Video level of agreement with face validity statements

**Face validity statements:**

1. The information in the video is easily understood by patients
2. The purpose of the patient information video is clear for patients
3. The video uses appropriate language and words for patients
4. Health terms and words are appropriately defined
5. The order of information in the video flows in a logical manner
6. Examples are used to bridge the gap between what patients know and what is being taught
7. The video breaks material into short sections
8. The video section titles are informative
9. The video is visually appealing (example: colour, heading, spacing)
10. The font or print size can be easily read by patients
11. Text on the screen is easy to read
12. The video uses pictures that are clear and uncluttered
13. The voice in the video is clear and easy to hear (not too fast, not garbled)
14. The content is presented in a style that is patient-centred so that the needs of the patients are most important
15. I would be confident using this patient information video

| **Overall levels of agreement per medication** | | | | | | | | | | | | | | | |
| --- | --- | --- | --- | --- | --- | --- | --- | --- | --- | --- | --- | --- | --- | --- | --- |
| **Alpha-1-Blockers** | | | | | | | | | | | | | | | |
| **Statement** | **1** | **2** | **3** | **4** | **5** | **6** | **7** | **8** | **9** | **10** | **11** | **12** | **13** | **14** | **15** |
| **Clinician Round** | 100% | 100% | 100% | 100% | 100% | 100% | 100% | 100% | 100% | 100% | 100% | 100% | 100% | 100% | 100% |
| **Round 1** | 83% | 83% | 83% | 83% | 83% | 50% | 83% | 83% | 83% | 67% | 67% | 67% | 67% | 67% | 83% |
| **Round 2** | 100% | 100% | 100% | 100% | 100% | 100% | 100% | 100% | 100% | 100% | 100% | 100% | 100% | 100% | 100% |
| **Round 3** | 67% | 83% | 83% | 83% | 83% | 83% | 100% | 100% | 100% | 100% | 100% | 100% | 83% | 100% | 100% |
| **Benzodiazepines / Z-Drugs** | | | | | | | | | | | | | | | |
| **Statement** | **1** | **2** | **3** | **4** | **5** | **6** | **7** | **8** | **9** | **10** | **11** | **12** | **13** | **14** | **15** |
| **Clinician Round** | 100% | 100% | 75% | 100% | 100% | 100% | 100% | 100% | 75% | 100% | 100% | 100% | 100% | 75% | 100% |
| **Round 1** | 83% | 100% | 100% | 100% | 100% | 100% | 100% | 100% | 83% | 100% | 100% | 100% | 100% | 100% | 100% |
| **Round 2** | 100% | 100% | 100% | 100% | 100% | 100% | 100% | 83% | 100% | 100% | 100% | 100% | 100% | 100% | 100% |
| **Round 3** | 83% | 100% | 67% | 83% | 100% | 100% | 100% | 100% | 100% | 100% | 100% | 100% | 100% | 100% | 100% |
| **Gabapentinoids** | | | | | | | | | | | | | | | |
| **Statement** | **1** | **2** | **3** | **4** | **5** | **6** | **7** | **8** | **9** | **10** | **11** | **12** | **13** | **14** | **15** |
| **Clinician Round** | 100% | 100% | 100% | 100% | 100% | 100% | 75% | 100% | 75% | 100% | 100% | 100% | 100% | 75% | 100% |
| **Round 1** | 100% | 100% | 100% | 100% | 100% | 100% | 100% | 100% | 83% | 100% | 100% | 100% | 100% | 100% | 100% |
| **Round 2** | 100% | 100% | 100% | 100% | 100% | 100% | 100% | 100% | 100% | 100% | 100% | 100% | 100% | 100% | 100% |
| **Round 3** | 100% | 100% | 100% | 100% | 100% | 100% | 100% | 100% | 100% | 100% | 100% | 83% | 100% | 100% | 100% |
| **Loop Diuretics** | | | | | | | | | | | | | | | |
| **Statement** | **1** | **2** | **3** | **4** | **5** | **6** | **7** | **8** | **9** | **10** | **11** | **12** | **13** | **14** | **15** |
| **Clinician Round** | 100% | 100% | 100% | 100% | 100% | 100% | 100% | 100% | 100% | 80% | 100% | 60% | 100% | 100% | 100% |
| **Round 1** | 100% | 100% | 100% | 100% | 100% | 100% | 83% | 83% | 100% | 100% | 100% | 100% | 83% | 100% | 83% |
| **Round 2** | 100% | 100% | 100% | 100% | 100% | 100% | 100% | 100% | 100% | 100% | 100% | 100% | 100% | 100% | 100% |
| **Round 3** | 100% | 100% | 100% | 100% | 100% | 100% | 100% | 100% | 100% | 100% | 100% | 100% | 100% | 100% | 100% |
| **Prokinetic Agents** | | | | | | | | | | | | | | | |
| **Statement** | **1** | **2** | **3** | **4** | **5** | **6** | **7** | **8** | **9** | **10** | **11** | **12** | **13** | **14** | **15** |
| **Clinician Round** | 100% | 100% | 80% | 100% | 100% | 100% | 100% | 100% | 100% | 100% | 100% | 100% | 100% | 100% | 100% |
| **Round 1** | 80% | 100% | 100% | 100% | 100% | 100% | 100% | 100% | 80% | 80% | 70% | 100% | 100% | 100% | 100% |
| **Round 2** | 100% | 100% | 100% | 100% | 100% | 100% | 100% | 100% | 100% | 100% | 100% | 80% | 100% | 100% | 100% |
| **Round 3** | 100% | 100% | 100% | 100% | 100% | 100% | 100% | 100% | 100% | 100% | 100% | 80% | 100% | 100% | 100% |
| **Proton Pump Inhibitors** | | | | | | | | | | | | | | | |
| **Statement** | **1** | **2** | **3** | **4** | **5** | **6** | **7** | **8** | **9** | **10** | **11** | **12** | **13** | **14** | **15** |
| **Clinician Round** | 100% | 100% | 80% | 80% | 100% | 100% | 100% | 100% | 100% | 80% | 100% | 100% | 80% | 80% | 100% |
| **Round 1** | 100% | 100% | 100% | 100% | 100% | 100% | 83% | 83% | 100% | 100% | 100% | 100% | 83% | 100% | 83% |
| **Round 2** | 100% | 100% | 100% | 100% | 100% | 100% | 100% | 100% | 100% | 100% | 100% | 100% | 100% | 100% | 100% |
| **Round 3** | 100% | 100% | 83% | 83% | 100% | 100% | 100% | 100% | 100% | 100% | 100% | 100% | 100% | 100% | 100% |
| **Quinines** | | | | | | | | | | | | | | | |
| **Statement** | **1** | **2** | **3** | **4** | **5** | **6** | **7** | **8** | **9** | **10** | **11** | **12** | **13** | **14** | **15** |
| **Clinician Round** | 100% | 100% | 100% | 50% | 75% | 100% | 100% | 100% | 100% | 75% | 75% | 75% | 100% | 100% | 100% |
| **Round 1** | 100% | 100% | 100% | 100% | 100% | 100% | 100% | 100% | 100% | 100% | 100% | 100% | 100% | 100% | 100% |
| **Round 2** | 100% | 83% | 83% | 100% | 83% | 100% | 100% | 83% | 67% | 83% | 100% | 83% | 100% | 83% | 100% |
| **Round 3** | 100% | 100% | 100% | 100% | 100% | 100% | 100% | 100% | 83% | 100% | 100% | 100% | 100% | 100% | 100% |
| **Statins** | | | | | | | | | | | | | | | |
| **Statement** | **1** | **2** | **3** | **4** | **5** | **6** | **7** | **8** | **9** | **10** | **11** | **12** | **13** | **14** | **15** |
| **Clinician Round** | 100% | 100% | 100% | 100% | 75% | 100% | 100% | 100% | 100% | 75% | 100% | 50% | 100% | 100% | 75% |
| **Round 1** | 100% | 100% | 100% | 100% | 100% | 100% | 100% | 100% | 100% | 100% | 100% | 100% | 100% | 100% | 100% |
| **Round 2** | 100% | 83% | 100% | 100% | 83% | 83% | 83% | 100% | 50% | 83% | 83% | 83% | 100% | 83% | 100% |
| **Round 3** | 100% | 100% | 100% | 100% | 100% | 83% | 83% | 100% | 83% | 100% | 100% | 100% | 100% | 100% | 100% |
| **Urate Lowering Agents** | | | | | | | | | | | | | | | |
| **Statement** | **1** | **2** | **3** | **4** | **5** | **6** | **7** | **8** | **9** | **10** | **11** | **12** | **13** | **14** | **15** |
| **Clinician Round** | 100% | 100% | 100% | 100% | 100% | 100% | 100% | 100% | 100% | 100% | 100% | 100% | 100% | 100% | 100% |
| **Round 1** | 67% | 83% | 83% | 83% | 83% | 67% | 83% | 83% | 83% | 67% | 67% | 83% | 67% | 67% | 83% |
| **Round 2** | 100% | 100% | 100% | 100% | 100% | 100% | 100% | 100% | 100% | 100% | 100% | 100% | 83% | 100% | 100% |
| **Round 3** | 83% | 83% | 83% | 83% | 100% | 100% | 100% | 100% | 100% | 100% | 100% | 100% | 83% | 100% | 100% |
| **General Deprescribing** | | | | | | | | | | | | | | | |
| **Statement** | **1** | **2** | **3** | **4** | **5** | **6** | **7** | **8** | **9** | **10** | **11** | **12** | **13** | **14** | **15** |
| **Round 1** | 100% | 100% | 100% | 100% | 100% | 83% | 100% | 100% | 100% | 100% | 100% | 100% | 100% | 100% | 100% |
| **Round 2** | 100% | 100% | 100% | 100% | 100% | 100% | 100% | 100% | 83% | 100% | 83% | 100% | 83% | 100% | 100% |
| **Round 3** | 100% | 100% | 100% | 100% | 100% | 83% | 100% | 100% | 83% | 100% | 100% | 100% | 100% | 83% | 83% |

# Appendix G: Summary of changes per validation round

Note: all changes were subsequently reflected in both bulletin and video, indicator states where the area of improvement was initially identified or deemed most prominent by reviewers.

## Changes made after initial clinician review

| **Item** | **Changes** |
| --- | --- |
| **Overall** | B: Add instruction to contact doctor or pharmacist regarding questions on dose reduction plan at the end of all bulletins  B: Improve consistency of lay terms used for providers: “doctor”, “pharmacist”, “nurse”  B: Emphasize medication restart allowed if patient changes mind |
| **Alpha-1-Blockers** | B: Improve lay explanation of drug indications  B: Add simple dietary suggestions; improved word consistency (i.e. stop vs. reduce); improved formatting consistency |
| **Benzodiazepines / Z-Drugs** | B: Replace “dependence” with “misuse”  B: Add lay explanation of “cognitive behavioural therapy”, lay rationale for other non-pharmaceutical options  B: Clarify need to notify all healthcare providers of discontinuation  V: Lessen number of listed benzodiazepine examples |
| **Gabapentinoids** | B: Alter wording to avoid stigmatization – change “abuse” to “misuse”, change “agitate” to “???”, remove term “drug seeking behaviour”; improve lay readability  B: Add non-pharmacological management strategies, more discontinuation risks  V: Change “outcome” to “patient care” |
| **Loop Diuretics** | B: Add more examples of common brand names; add indication benign prostate hyperplasia  V: Rephrased “Restart your loop diuretic at previous dose” to “Restart your loop diuretic” to reflect realistic care scenario if drug restart is needed |
| **Prokinetic Agents** | B: Alter wording for clarity: “defecate” to “bowel movement” |
| **Proton Pump Inhibitors** | B: Add phrase “or any foods that worsen your symptoms” to examples of heartburn triggering foods  V: Clarify nature of PPI cardiac risks to avoid unnecessary alarm |
| **Quinines** | B: Add non-pharmacological management strategies, discontinuation risks; clarified explanation of side effects  B: Add explanation of tapering using pills/capsules as unit  V: Non-pharmacological alternatives presented before pharmacological alternatives |
| **Statins** | B: Clarify monitoring plan, lay explanation of management strategies  V: Clarify symptom monitoring explanation |
| **Urate Lowering Agents** | B: Replace “uric acid” with “gout attacks”, simplify explanation of uric acid crystal deposits to lay language  B: Add emphasis on risks of discontinuation, recommendation to consult dietitian |

B = bulletin; V = video

## Changes made after first patient review round

| **Item** | **Changes** |
| --- | --- |
| **Overall** | B: Add emphasis to notify healthcare providers if symptoms return  B: Add placeholder area for a QR code to the final video  V: Slowed pacing of narration and transitions  B: Reviewed medical terminology to increase lay readability |
| **Deprescribing (General)** | A bulletin and video were created after feedback from this review round, with the following goals:   - Explain polypharmacy and risks; introduce deprescribing, how it can lower polypharmacy risk - Align with patient attitudes: less emphasis on deprescribing rationale in HD, lack of HD patients in clinical trials - Add clarity that deprescribing is about balancing harms and benefits   *Deprescribing (general) bulletin and video had no clinician round; underwent 3 patient rounds directly |
| **Benzodiazepines / Z-Drugs** | B: Shorten headings to avoid splitting words between lines  B: Add formatting to subheadings for emphasis and clarity |
| **Gabapentinoids** | B: Shorten headings to avoid splitting words between lines  B: Add formatting to subheadings for emphasis and clarity |
| **Prokinetic Agents** | B: Take “domperidone” and “metoclopramide” out of title and add section on examples of these medications. |
| **Proton Pump Inhibitors** | B: Corrected error in section title |
| **Quinines** | B: Reorder quinine side effects, emphasize cardiac risk; patients may not see cardiac risks and choose to ignore as cramps are painful |
| **Urate Lowering Agents** | B: Add explanation of uric acid in the body, relevance to gout attacks |

B = bulletin; V = video

## Changes made after second patient review round

| **Item** | **Changes** |
| --- | --- |
| **Overall** | B: Add tapering dose schematic where applicable to help patients understand tapering process |
| **Quinines** | B: Merge “Why reduce dose or stop your quinine dose?” and “What are the safety concerns of taking quinines?”, as presented information is identical |
| **Statins** | B: Add emphasis of ongoing clinical monitoring for labs and symptoms |

B = bulletin; V = video

## Changes made after the third patient review round

| **Item** | **Changes** |
| --- | --- |
| **Overall** | No changes made  Deprescribing (general) bulletin and video had no major revisions |
