# Supplementary material for: Development and Validation of Patient Education Tools for Deprescribing in Patients on Hemodialysis
Source: Can J Kidney Health Dis. 2023 Jan 24;10:20543581221150676. doi: 10.1177/20543581221150676 (PMC9880575; doi:10.1177/20543581221150676)

Appendix II Contents

[Appendix H: Deprescribing Bulletin for Alpha_1_-Blockers 2](#_Toc112770219)

[Appendix I: Deprescribing Bulletin for Benzodiazepine & Z-drugs 4](#_Toc112770220)

[Appendix J: Deprescribing Bulletin for Gabapentinoids 6](#_Toc112770221)

[Appendix K: Deprescribing Bulletin for Loop Diuretics 8](#_Toc112770222)

[Appendix L: Deprescribing Bulletin for Prokinetic Agents 10](#_Toc112770223)

[Appendix M: Deprescribing Bulletin for Proton Pump Inhibitors (PPI) 12](#_Toc112770224)

[Appendix N: Deprescribing Bulletin for Quinine 14](#_Toc112770225)

[Appendix O: Deprescribing Bulletin for Statins 16](#_Toc112770226)

[Appendix P: Deprescribing Bulletin for Urate Lowering Agents 18](#_Toc112770227)

[Appendix Q: General Deprescribing Bulletin 20](#_Toc112770228)

# Appendix H: Deprescribing Bulletin for Alpha_1_-Blockers


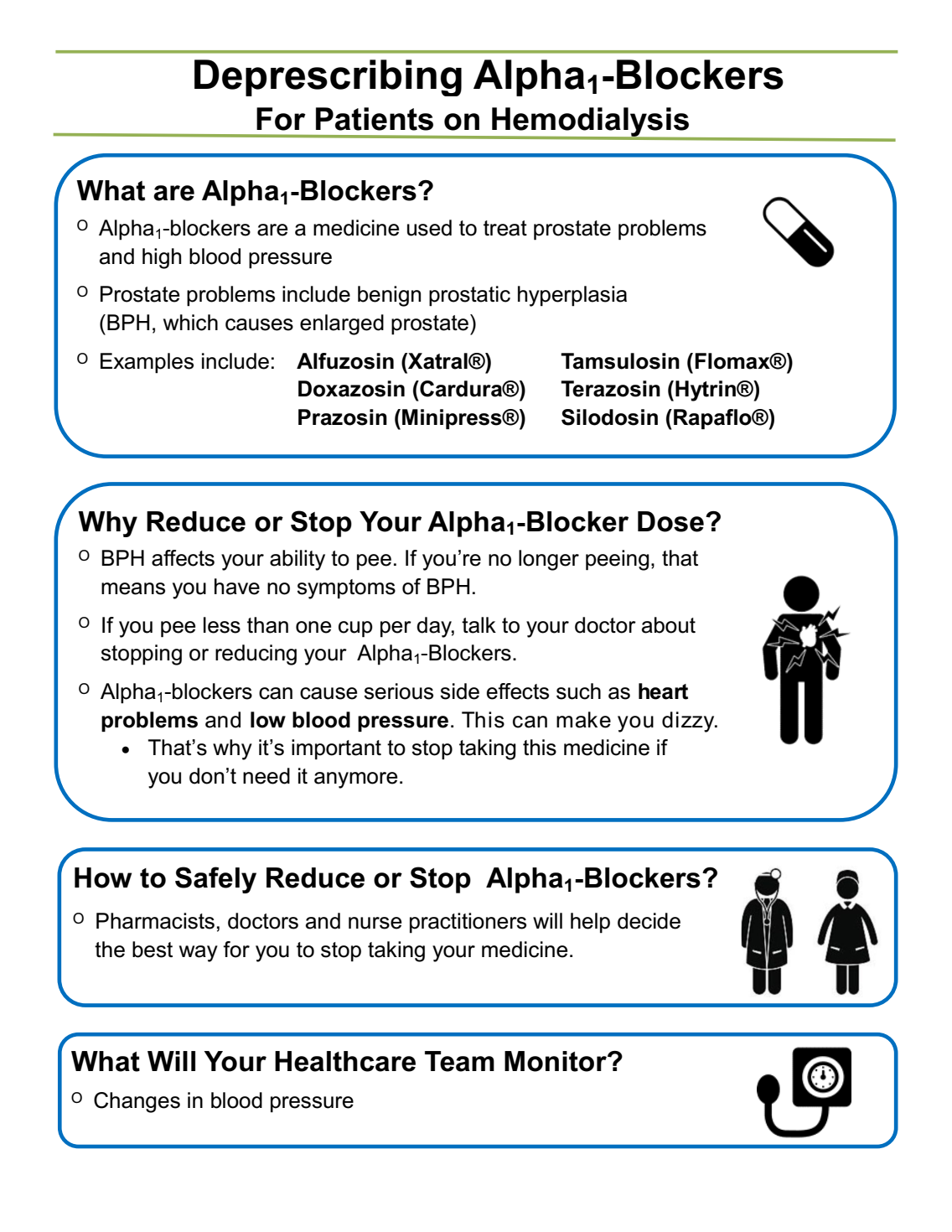


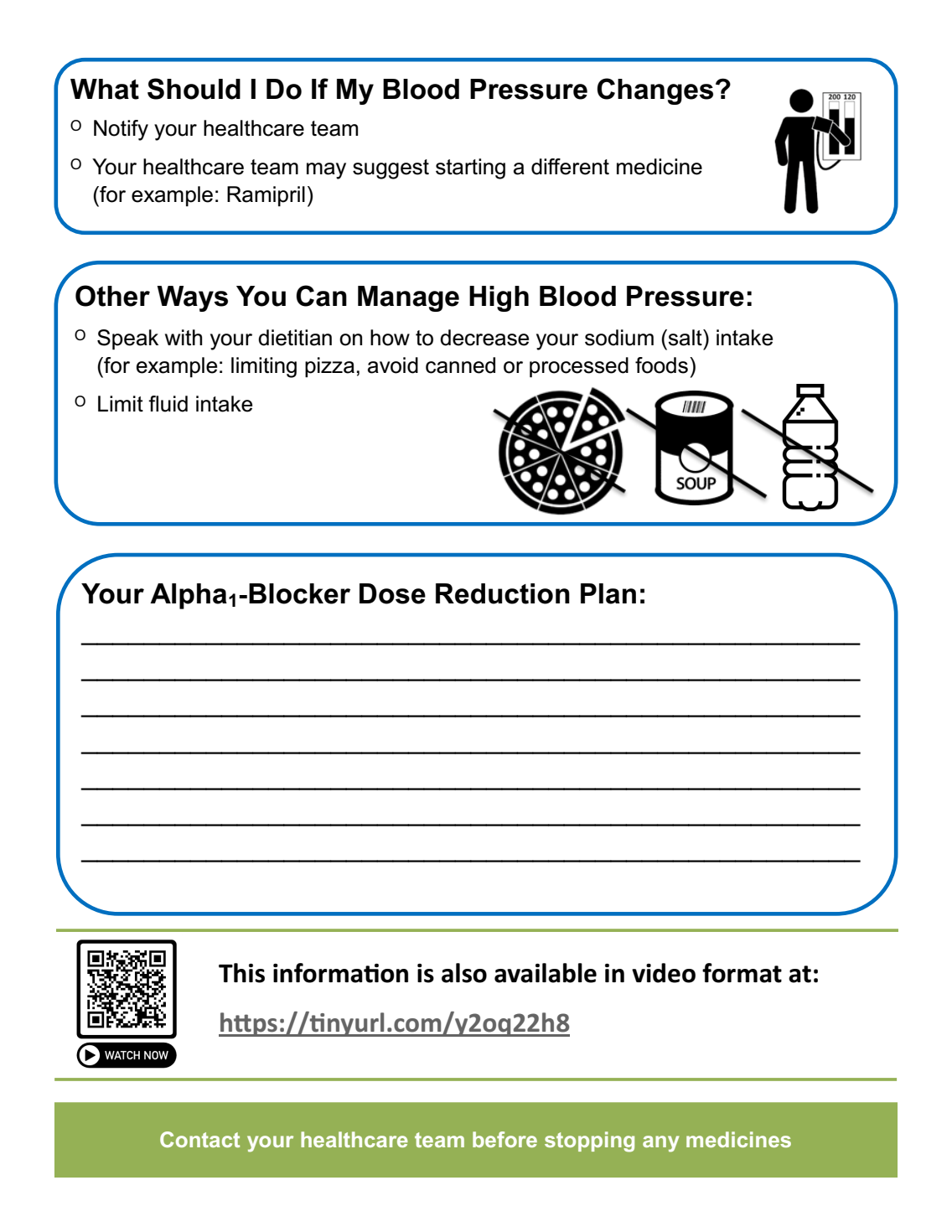


# Appendix I: Deprescribing Bulletin for Benzodiazepine & Z-drugs


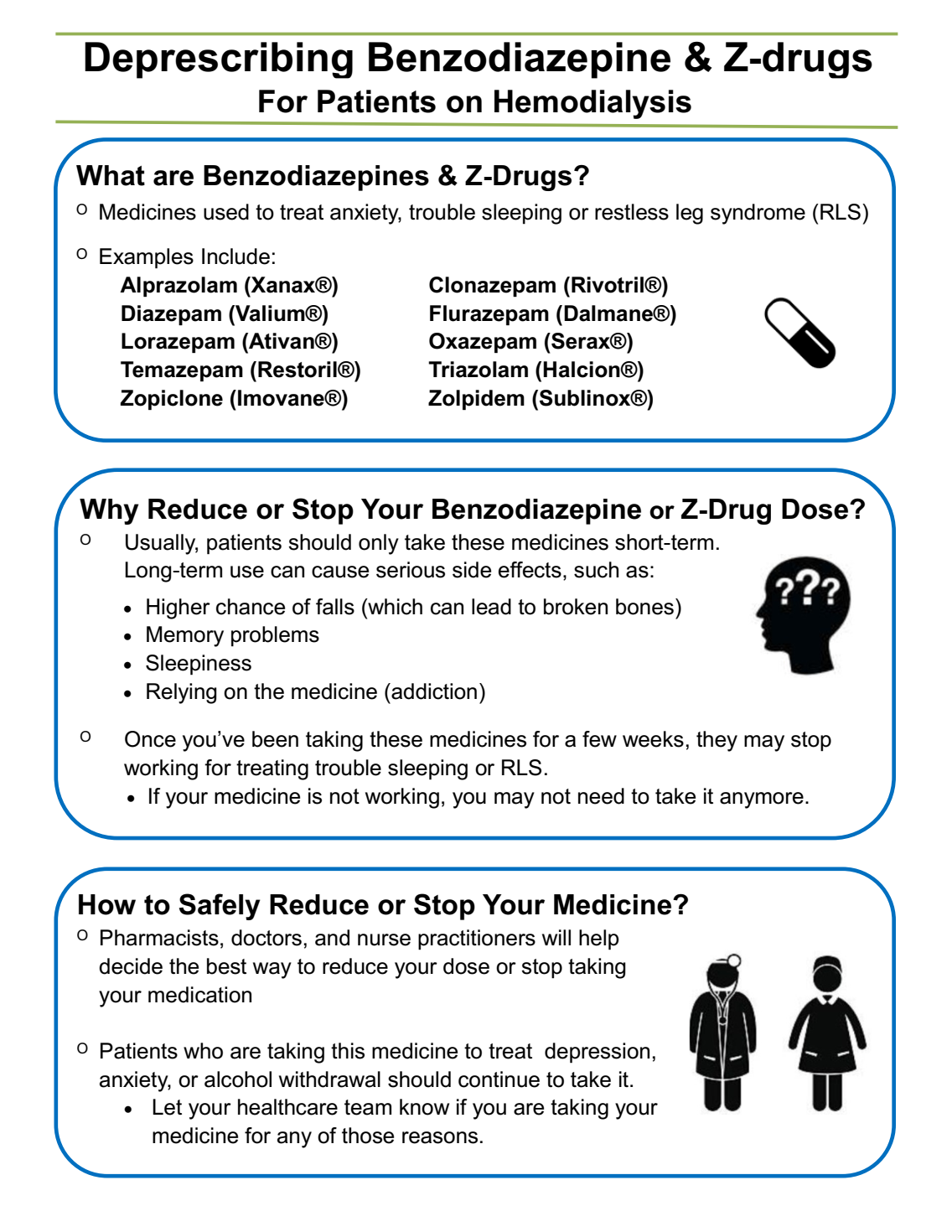


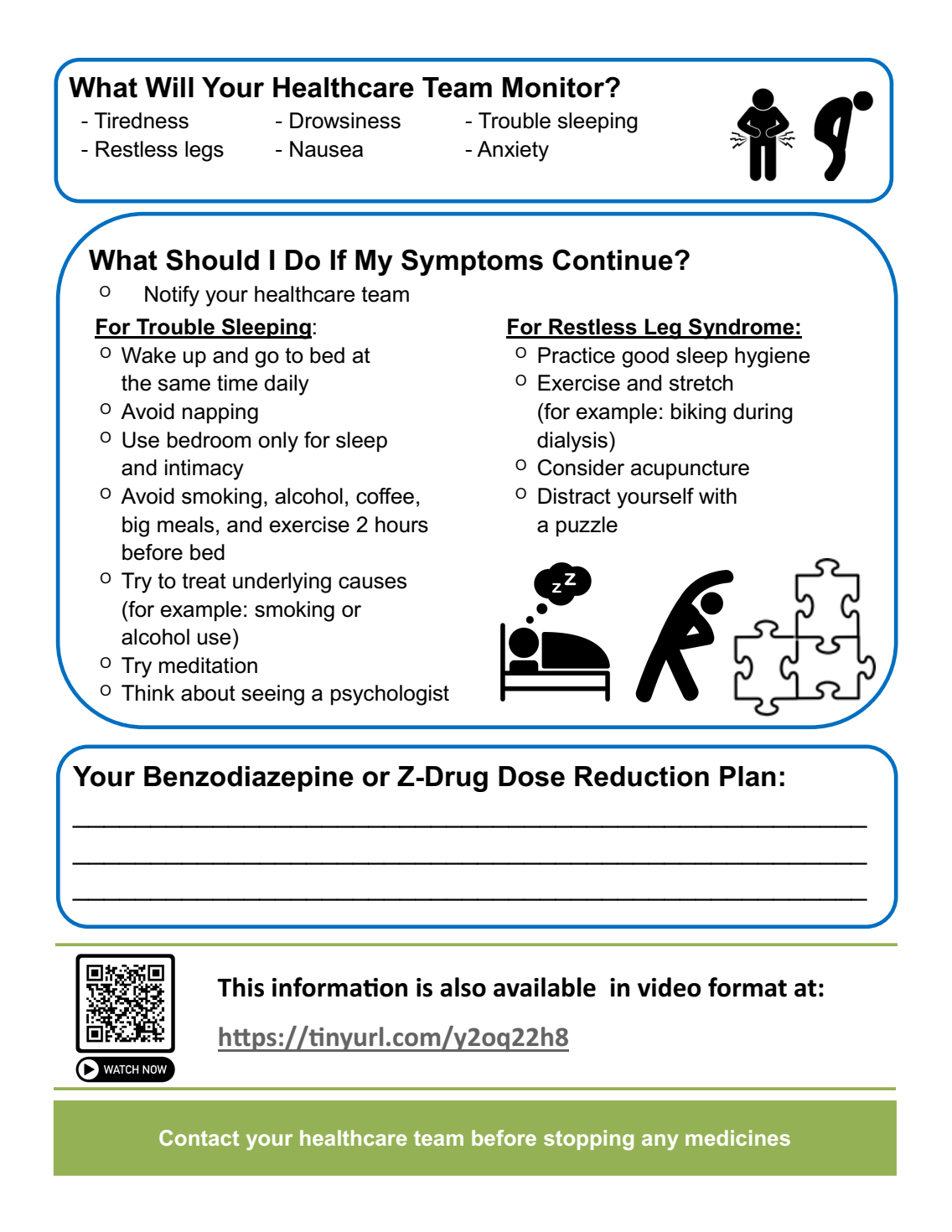


# Appendix J: Deprescribing Bulletin for Gabapentinoids


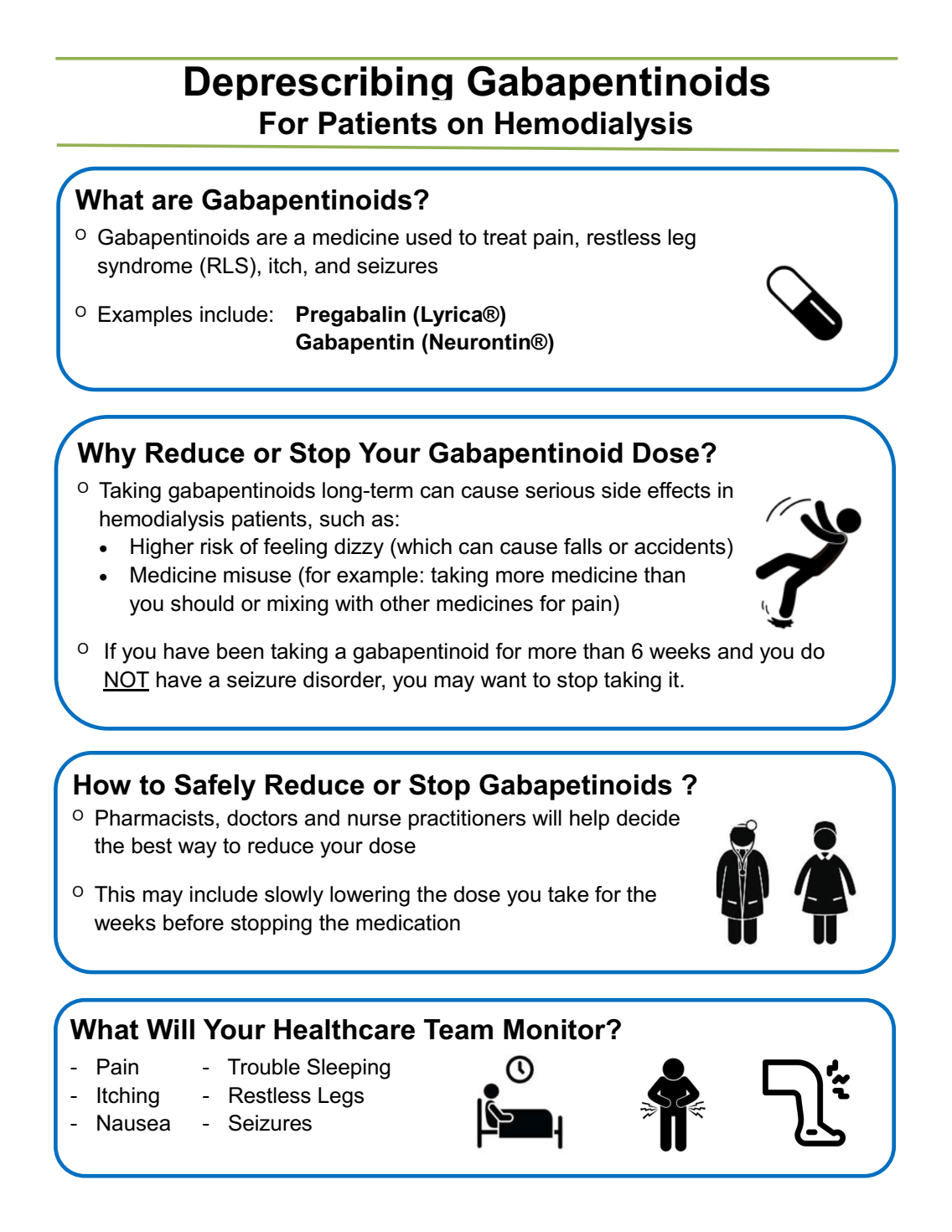


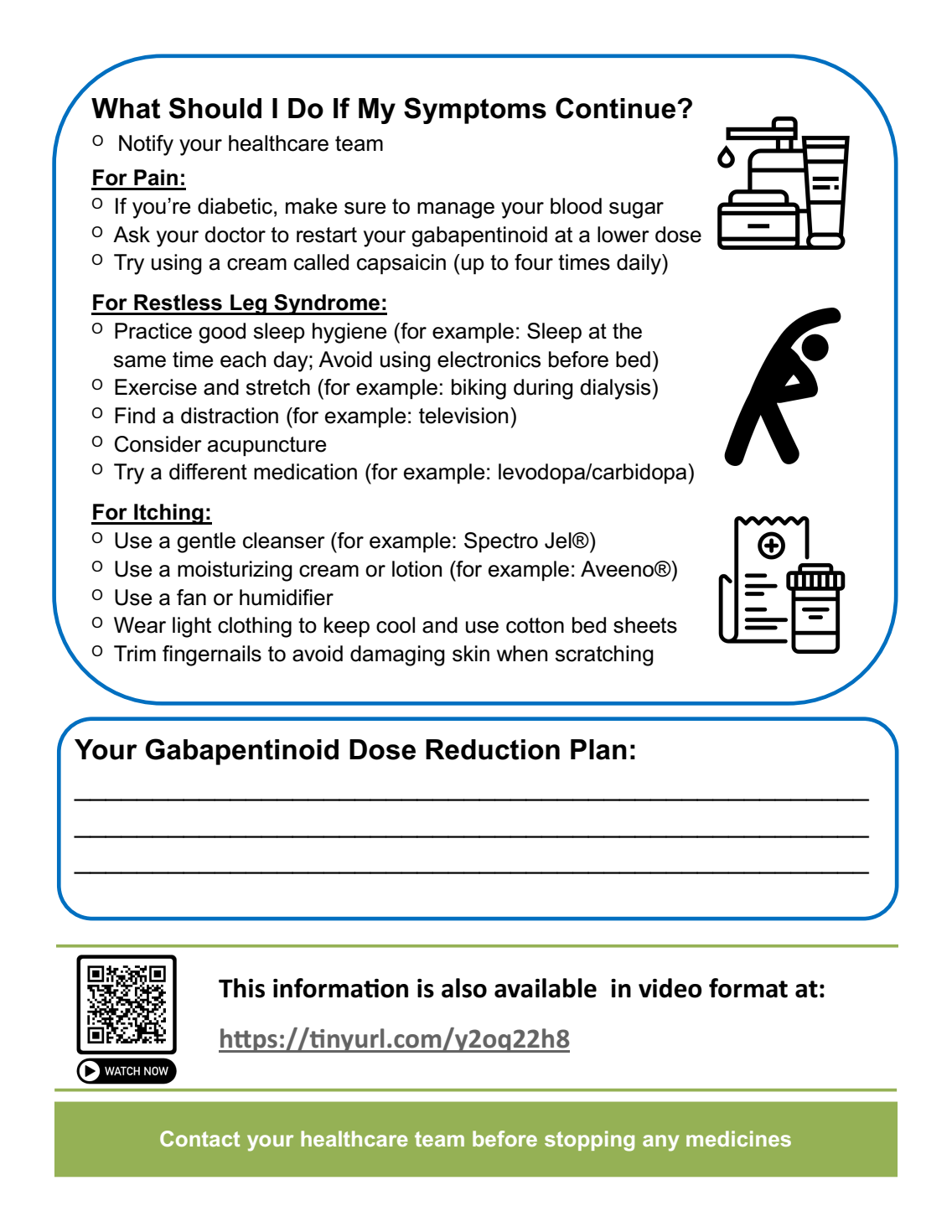


# Appendix K: Deprescribing Bulletin for Loop Diuretics


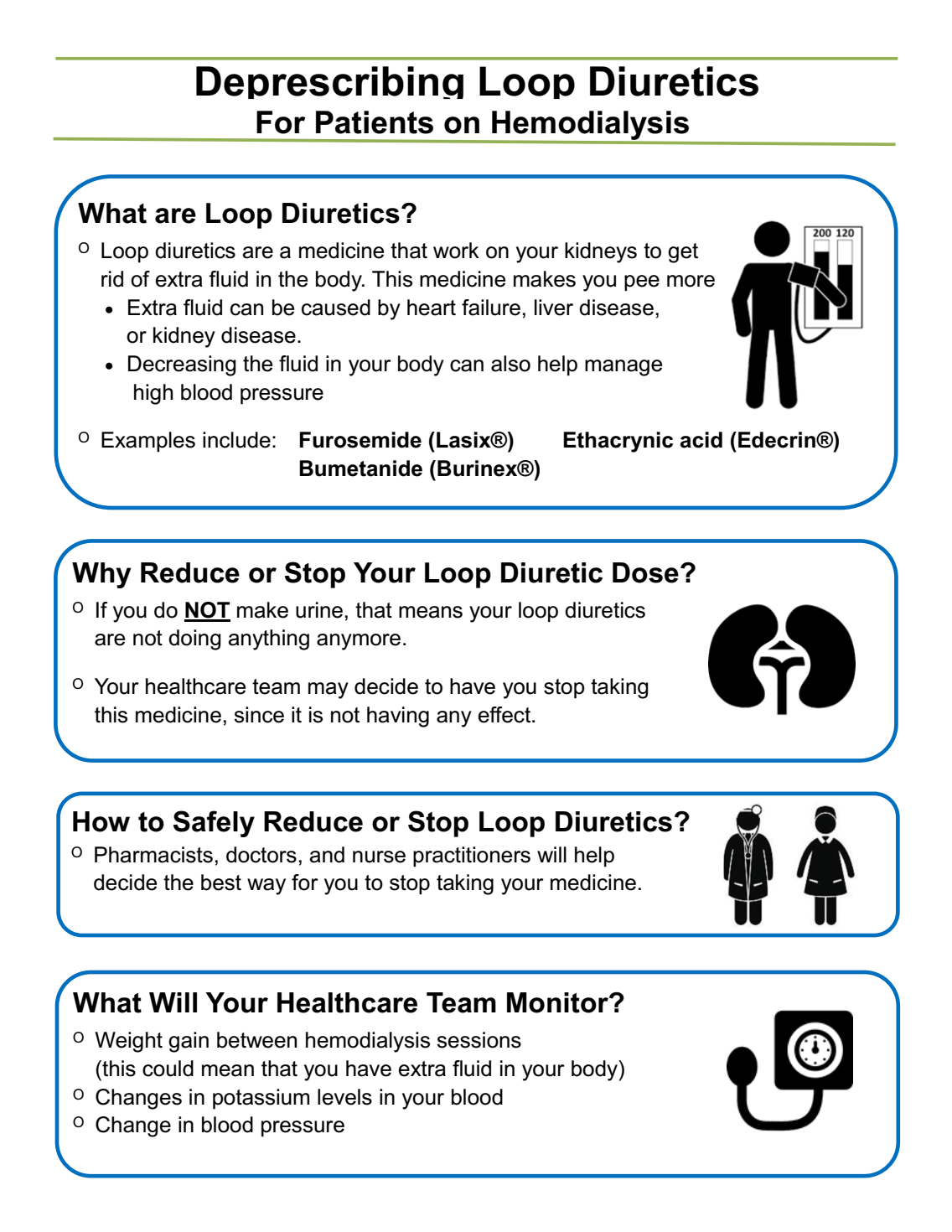


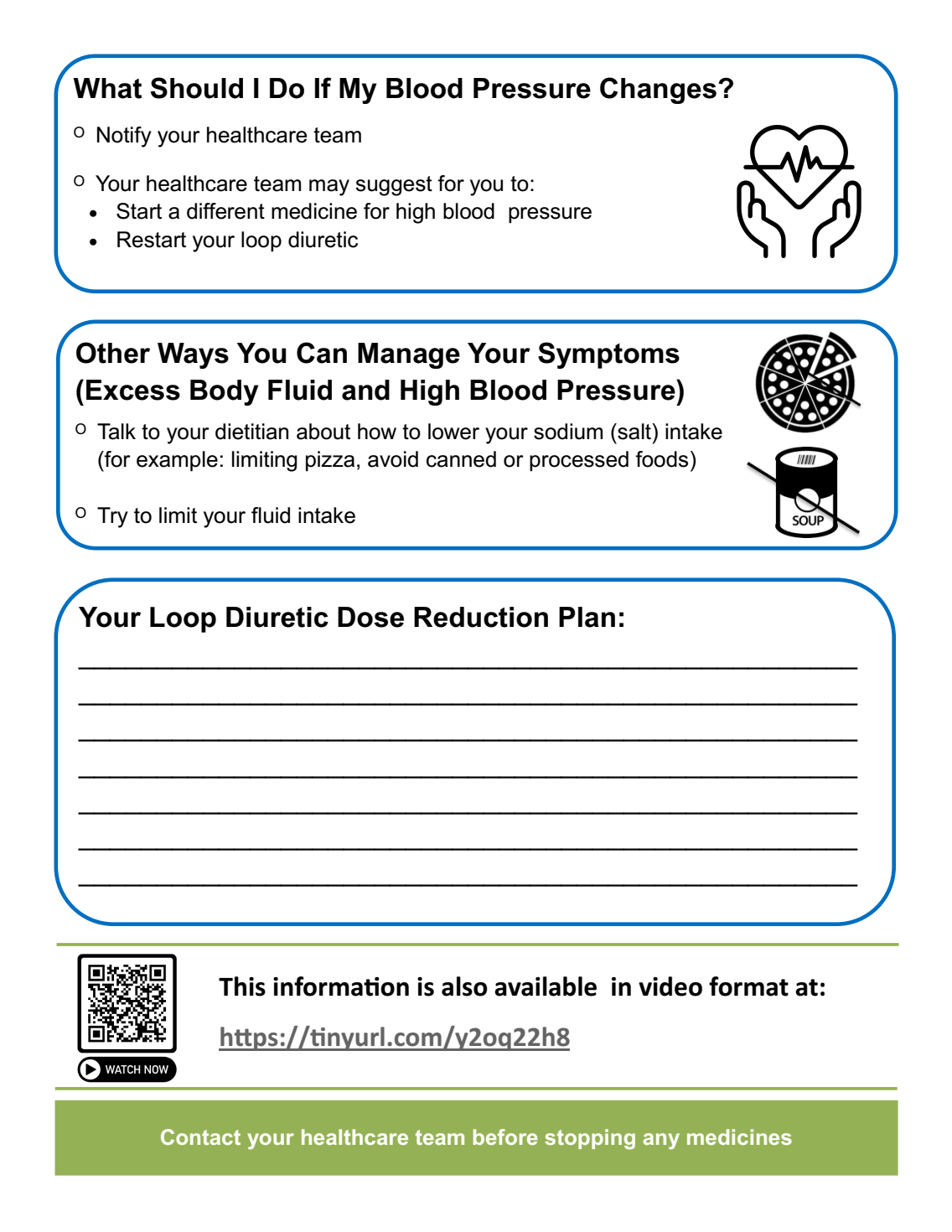


# Appendix L: Deprescribing Bulletin for Prokinetic Agents


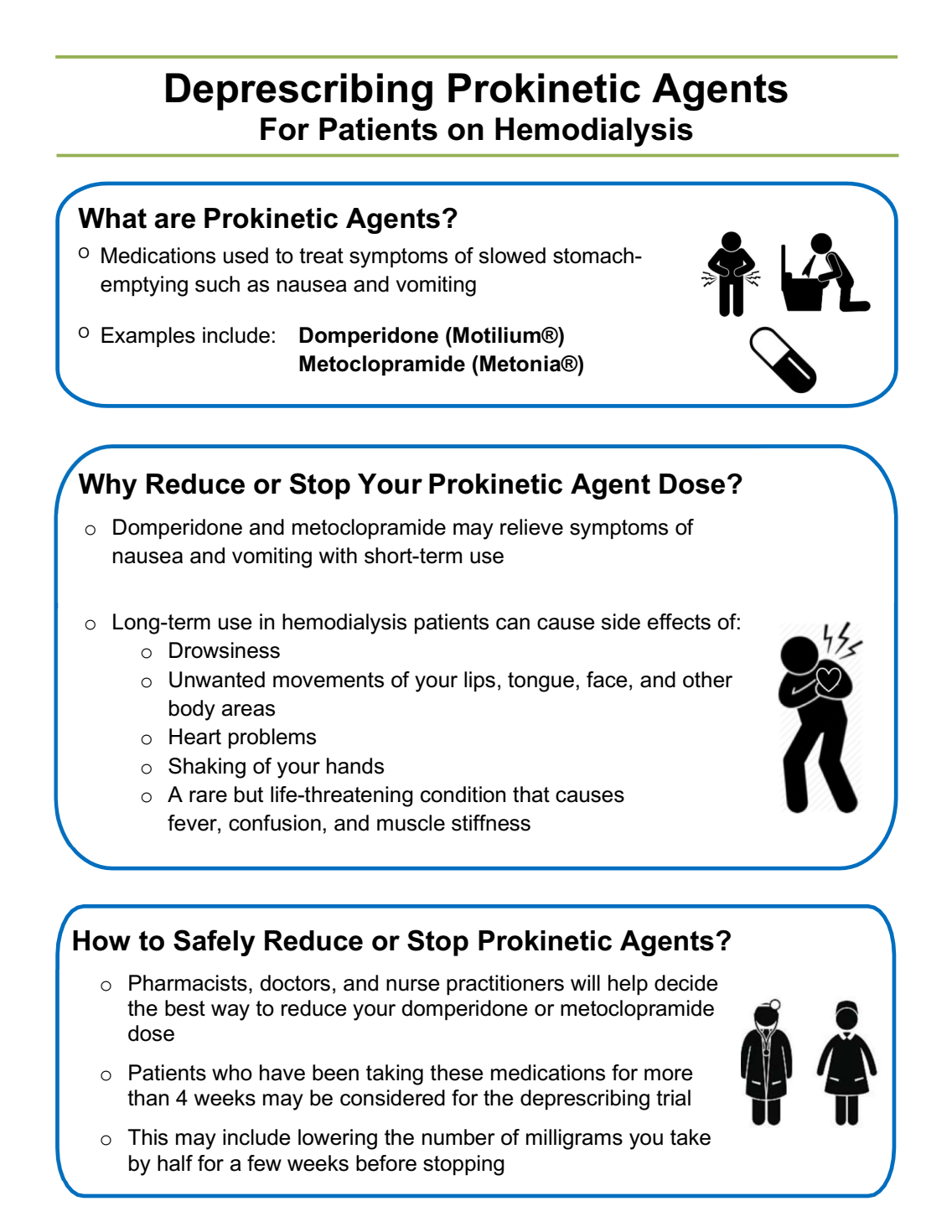


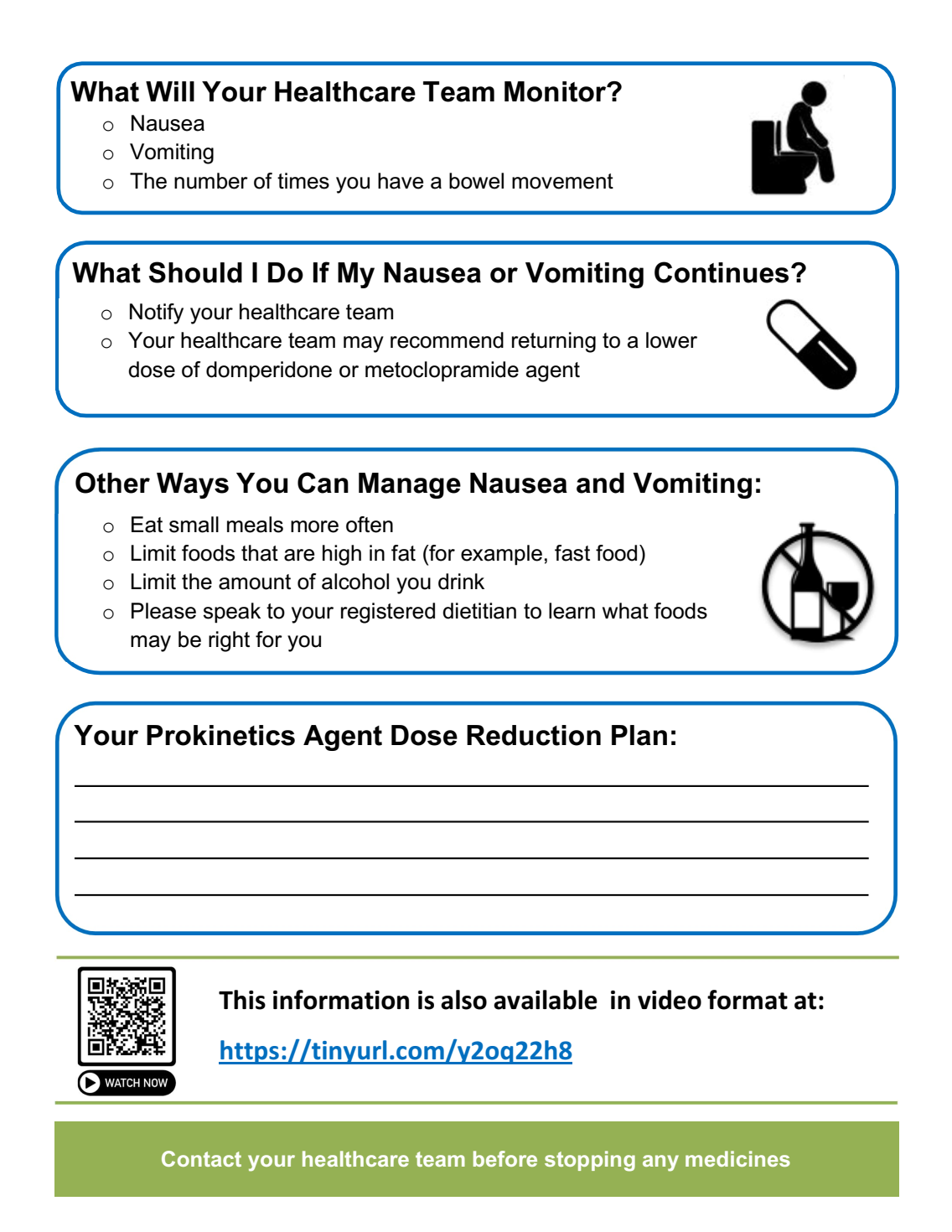


# Appendix M: Deprescribing Bulletin for Proton Pump Inhibitors (PPI)


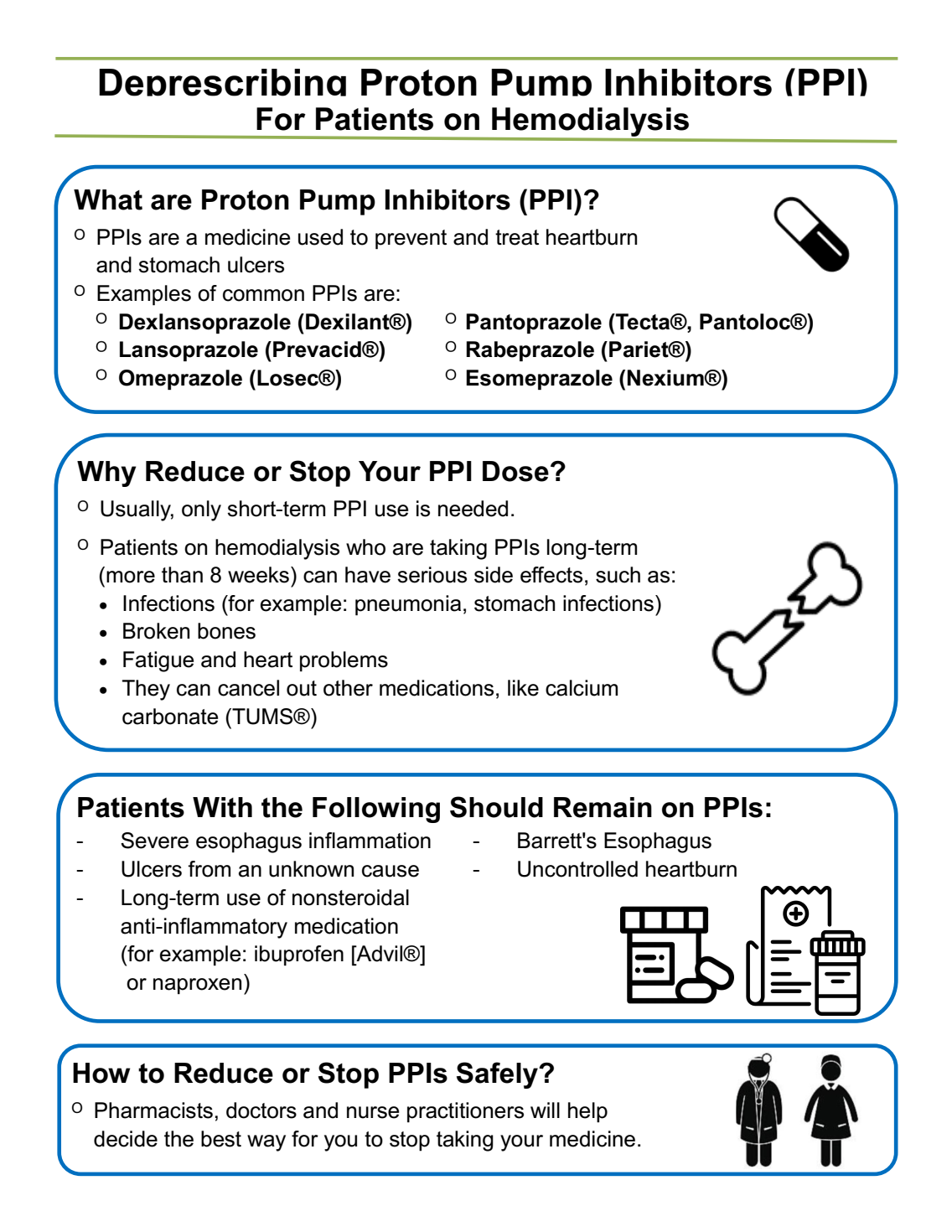


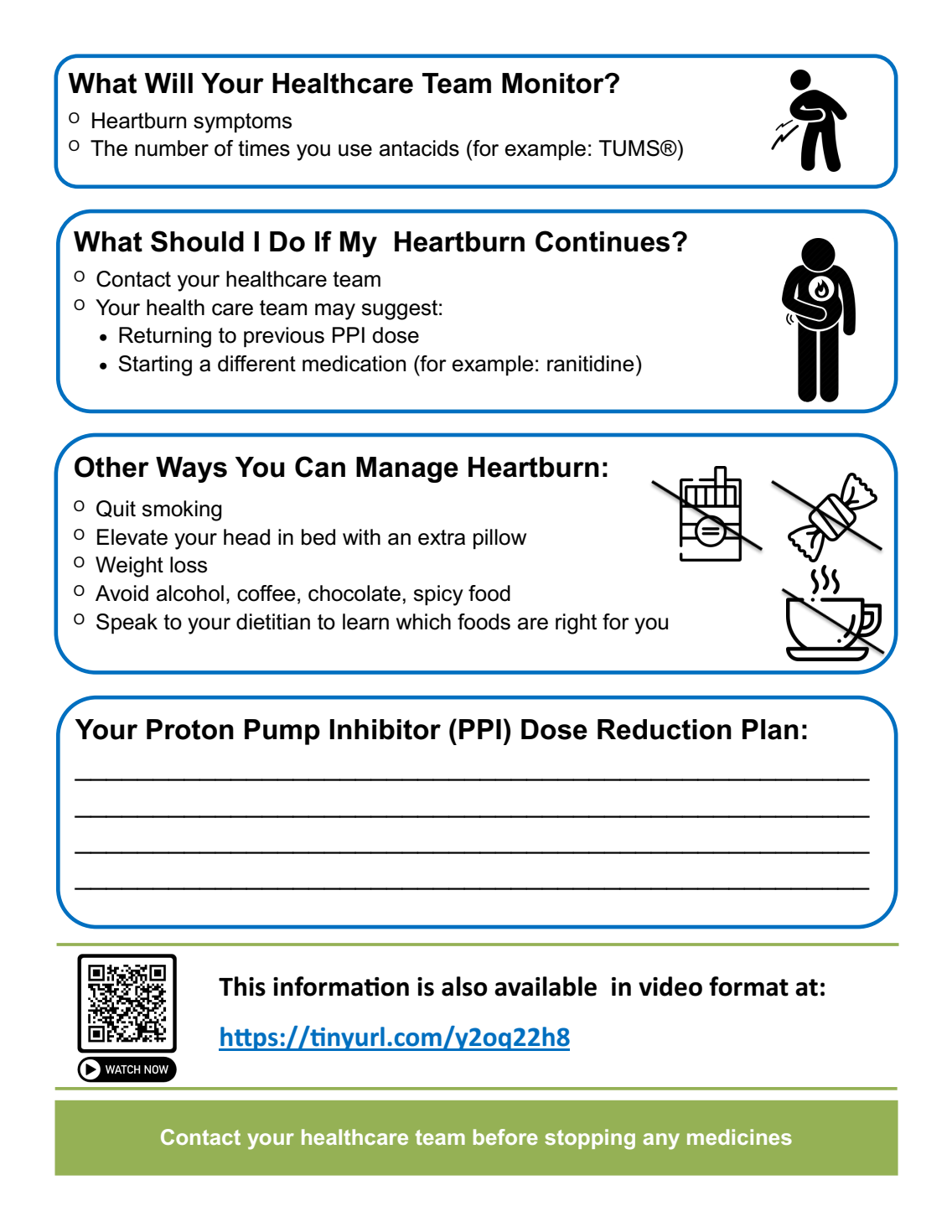


# Appendix N: Deprescribing Bulletin for Quinine


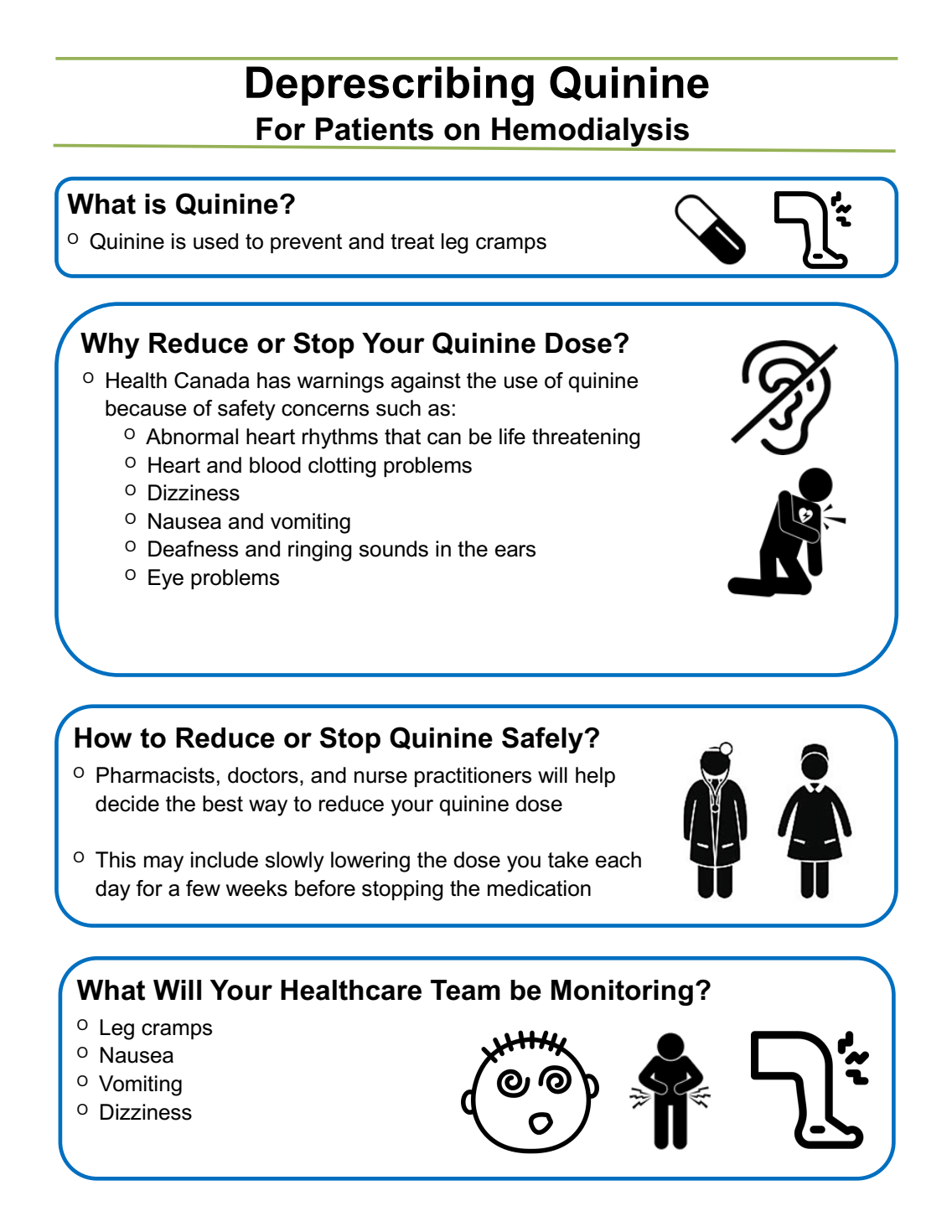


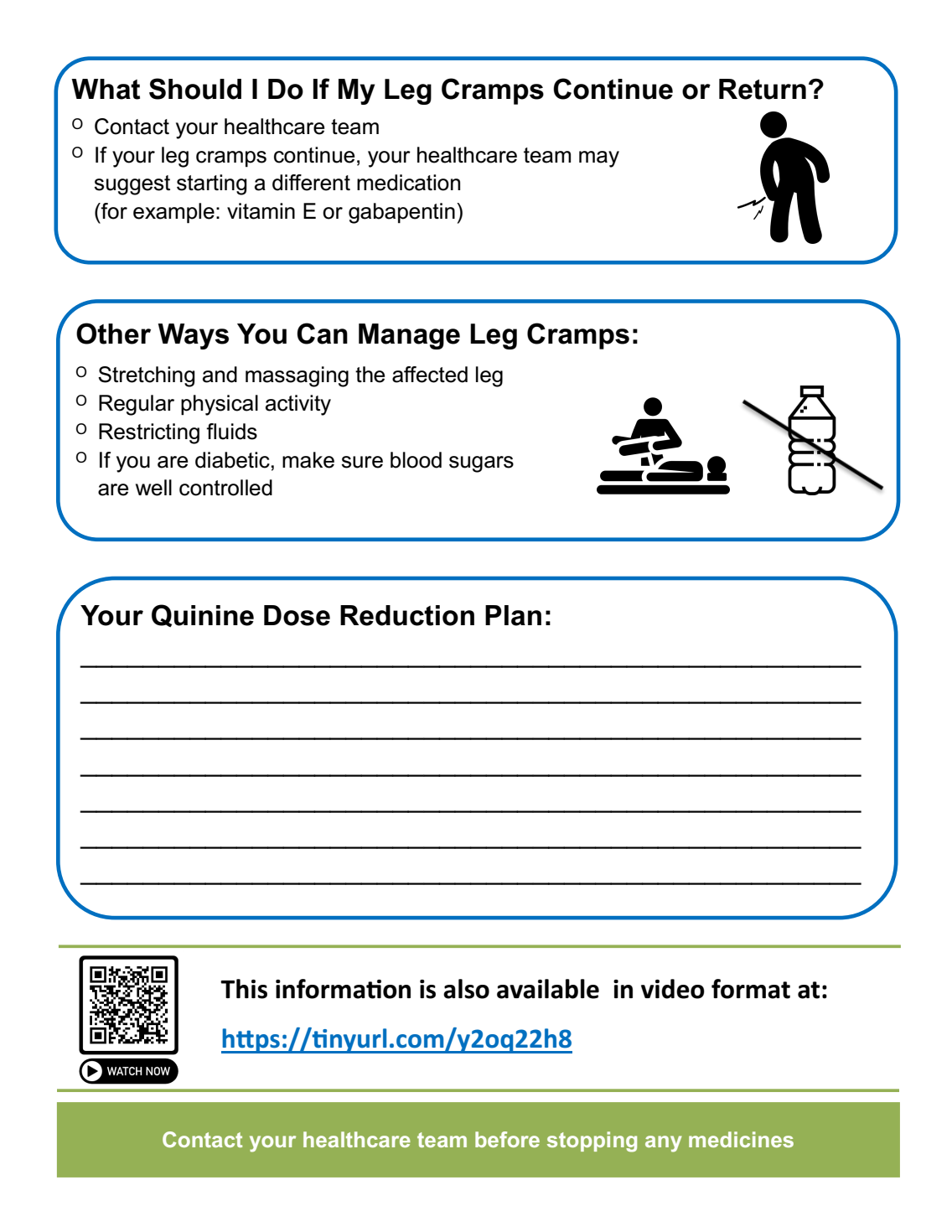


# Appendix O: Deprescribing Bulletin for Statins


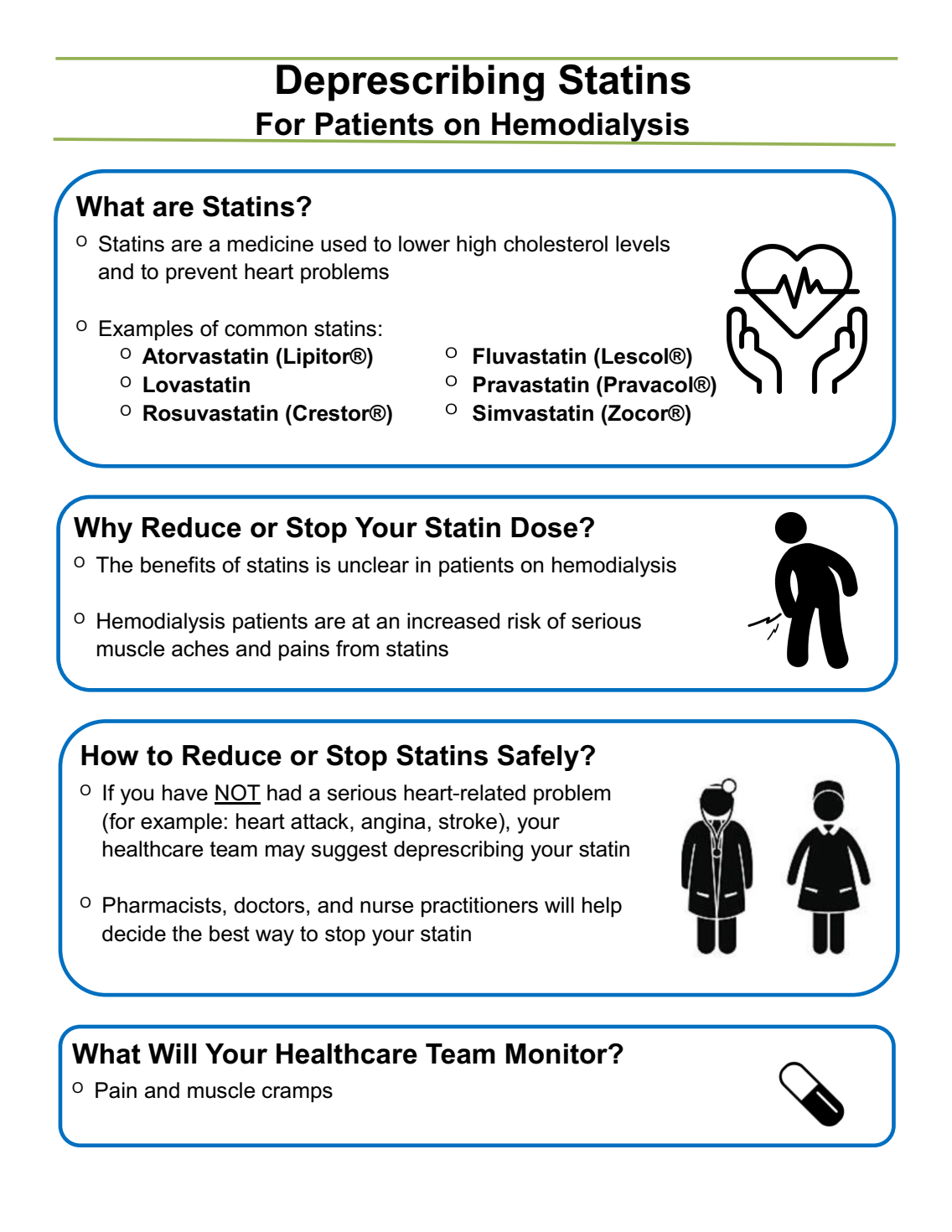


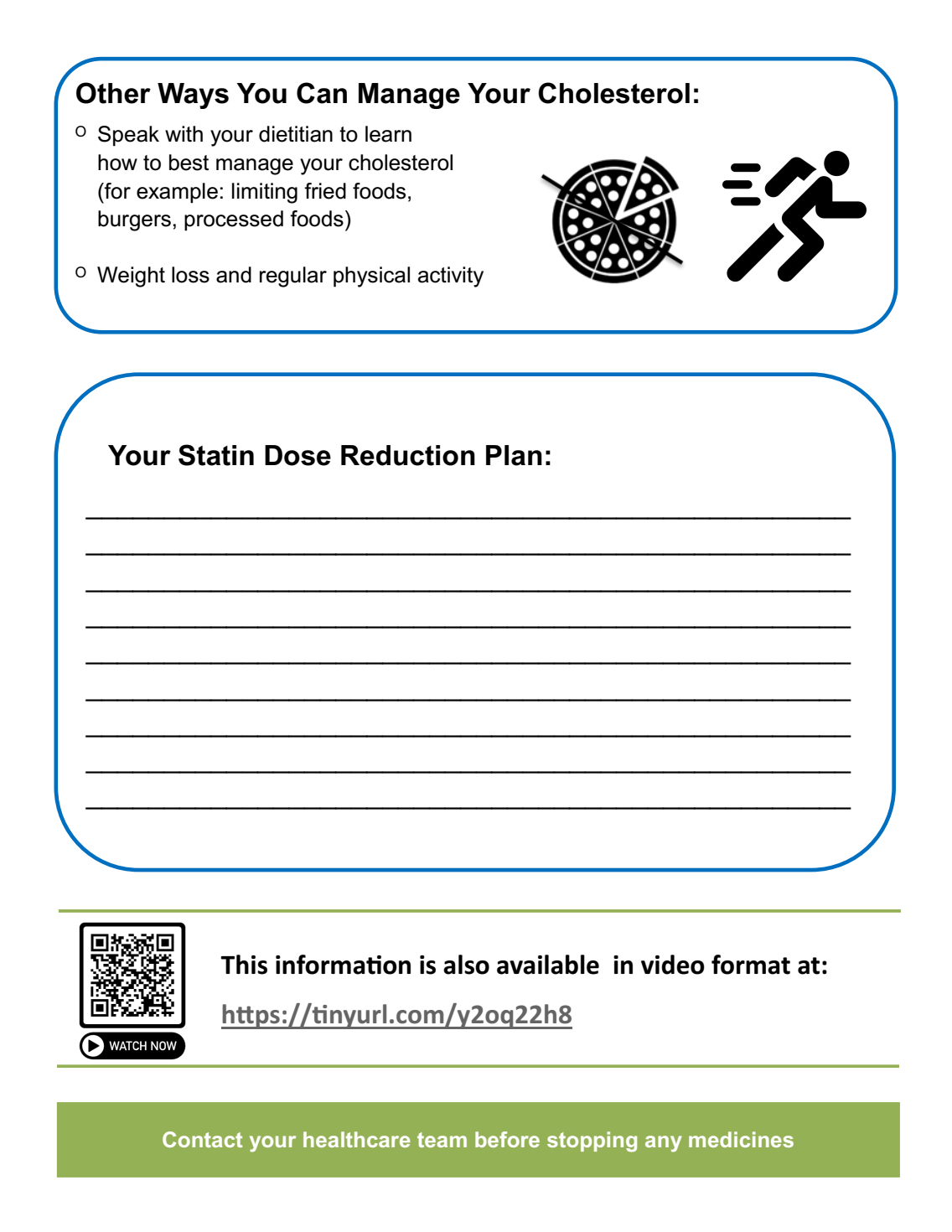


# Appendix P: Deprescribing Bulletin for Urate Lowering Agents


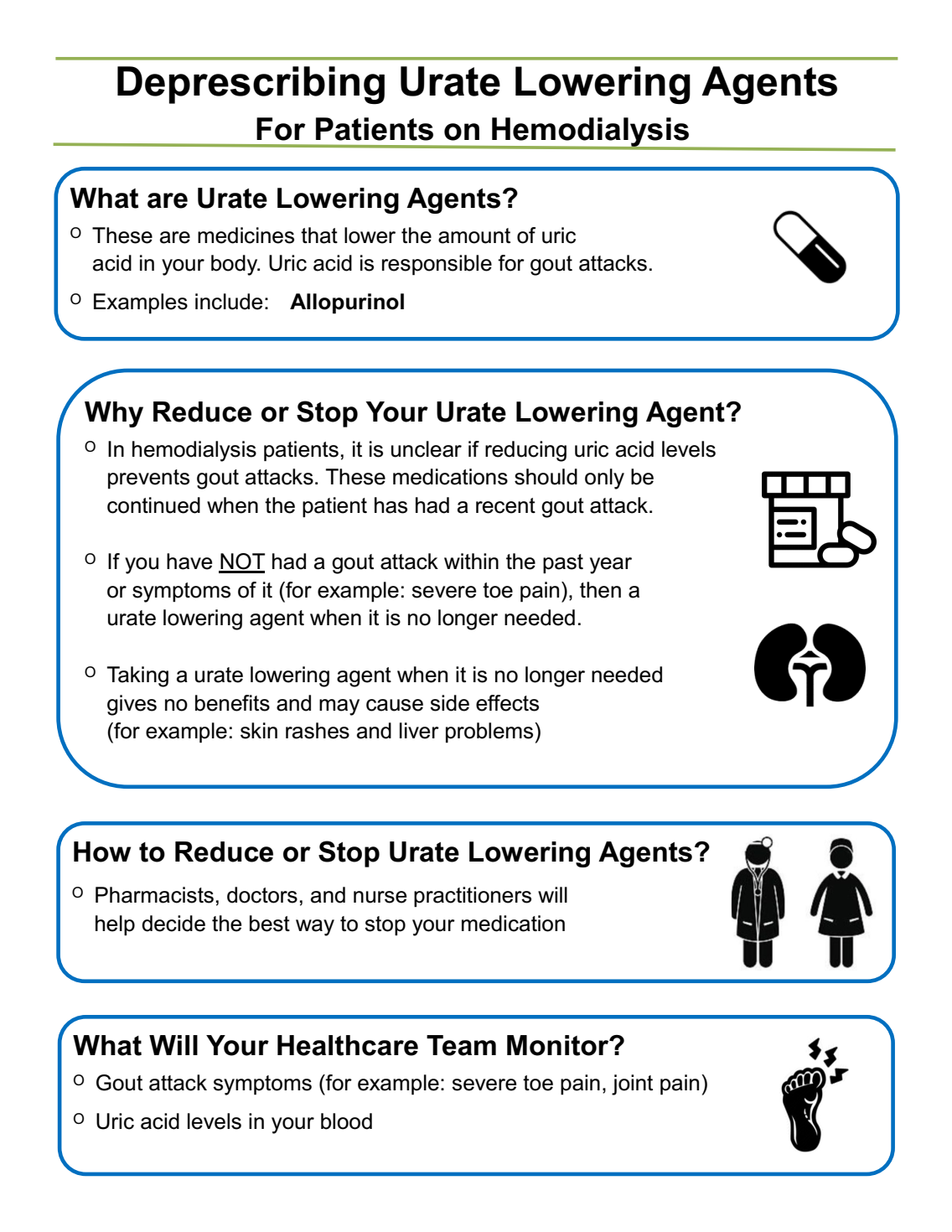


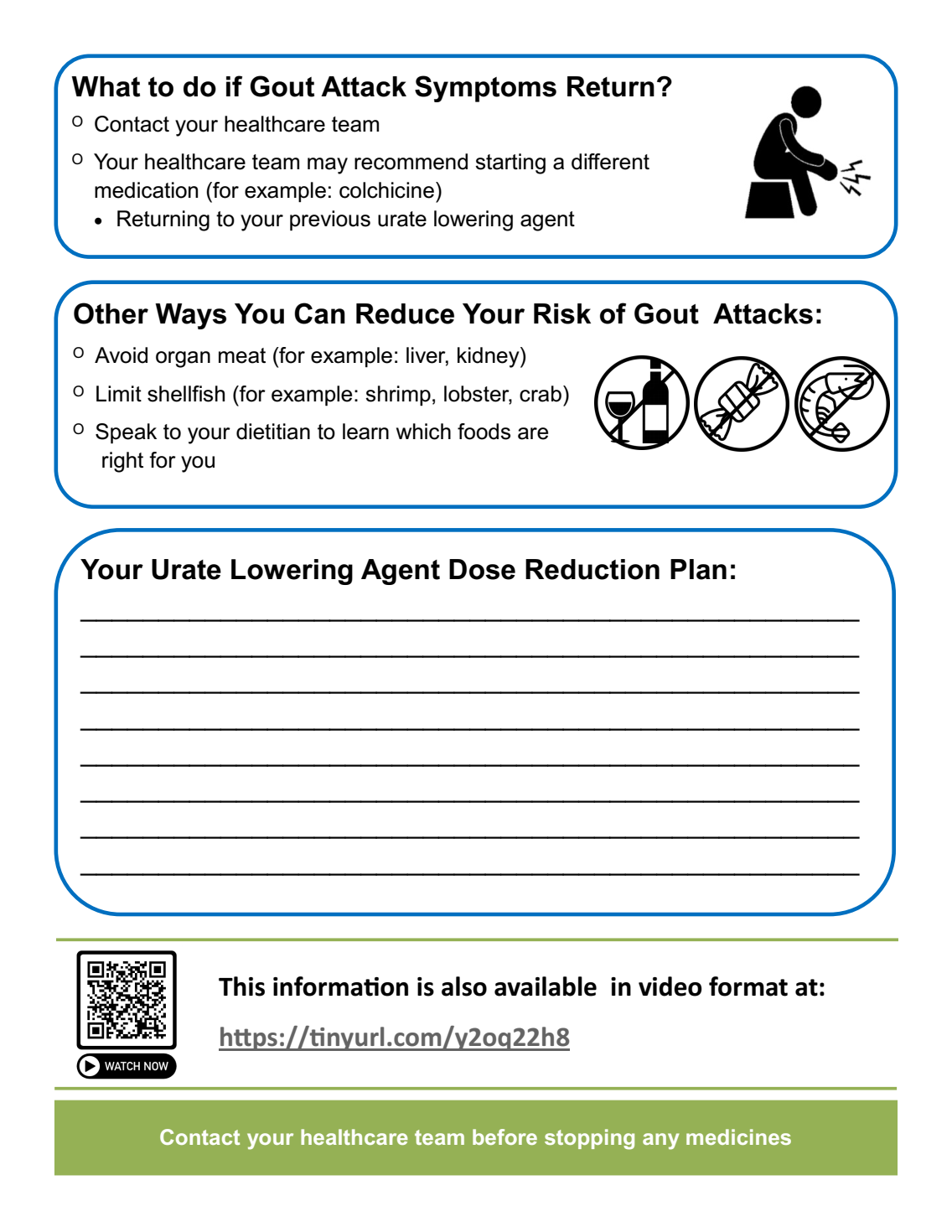


# Appendix Q: General Deprescribing Bulletin


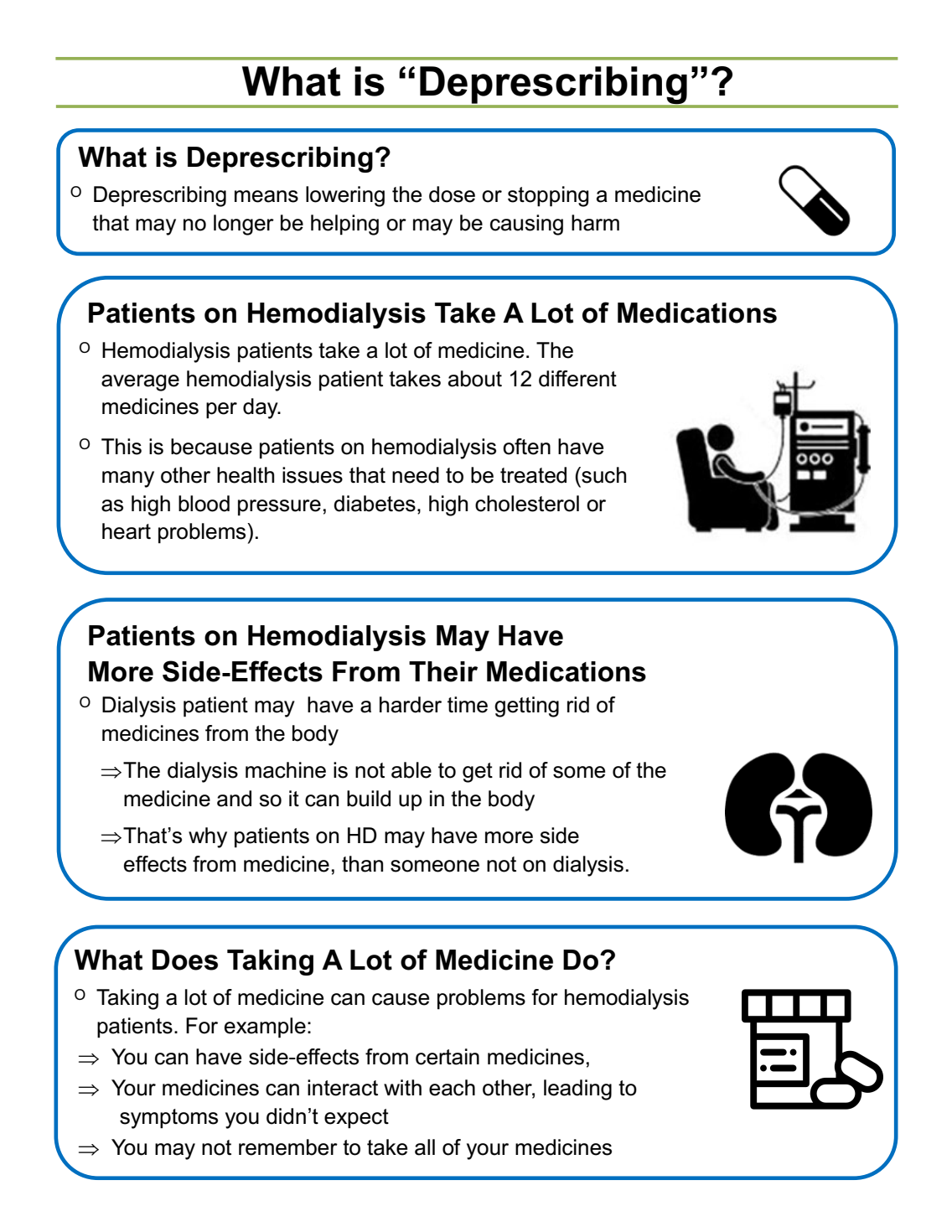


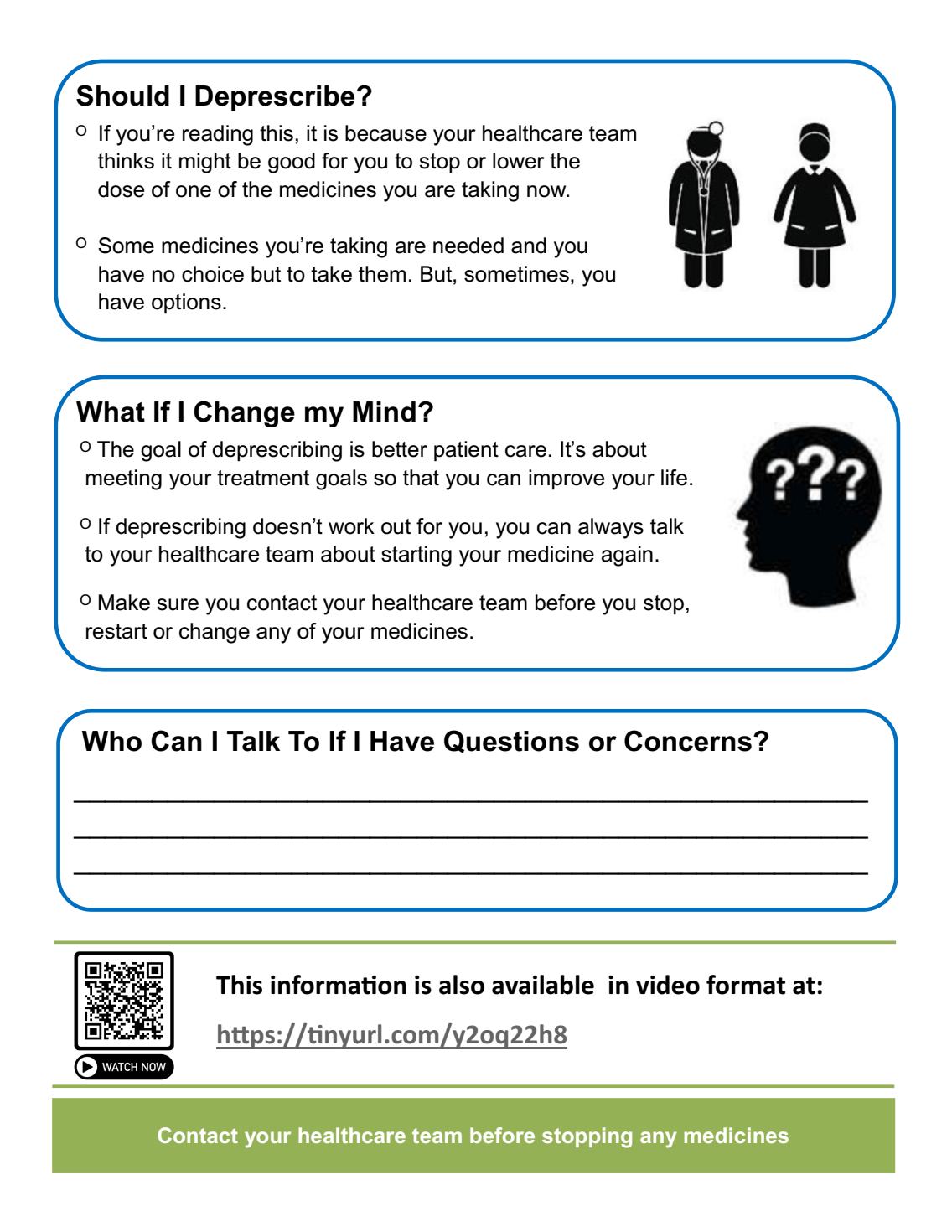

Supplement: sj-docx-2-cjk-10.1177_20543581221150676 – Supplemental material for Development and Validation of Patient Education Tools for Deprescribing in Patients on Hemodialysis [file sj-docx-2-cjk-10.1177_20543581221150676.docx]
